# Chromatographic Fingerprinting of the Old World Lupins Seed Alkaloids: A Supplemental Tool in Species Discrimination

**DOI:** 10.3390/plants8120548

**Published:** 2019-11-27

**Authors:** Wojciech Święcicki, Katarzyna Czepiel, Paulina Wilczura, Paweł Barzyk, Zygmunt Kaczmarek, Magdalena Kroc

**Affiliations:** 1 Department of Genomics, Institute of Plant Genetics, Polish Academy of Sciences, Strzeszyńska 34, 60-479 Poznan, Poland; wswi@igr.poznan.pl (W.Ś.); kcze@igr.poznan.pl (K.C.); mwil@igr.poznan.pl (P.W.); 2 Poznan Plant Breeders Ltd., Wiatrowo Plant Breeding Branch, 62-100 Wengrowitz, Poland; pawel.barzyk@phr.pl; 3 Department of Biometry and Bioinformatics, Institute of Plant Genetics, Polish Academy of Sciences, Strzeszyńska 34, 60-479 Poznan, Poland; zkac@igr.poznan.pl

**Keywords:** alkaloid profiles, species discrimination, Old World lupins, gas chromatography, *Lupinus*, valorization of genetic resources

## Abstract

The total contents and qualitative compositions of alkaloids in seeds of 10 Old World lupin species (73 accessions) were surveyed using gas chromatography. The obtained results, combined with those for three lupin crops, *Lupinus angustifolius*, *Lupinus albus*, and *Lupinus luteus*, provide the most complete and up-to-date overview of alkaloid profiles of 13 lupin species originating from the Mediterranean Basin. The qualitative alkaloid compositions served as useful supplementary tools of species discrimination. On the basis of the most abundant major alkaloids, lupanine, lupinine, and multiflorine, the Old World lupin species were divided into four groups. Those containing lupanine (*L. angustifolius*, *L. albus*, and *Lupinus mariae-josephi*), containing lupinine (*Lupinus luteus*, *Lupinus hispanicus*, and *Lupinus* × *hispanicoluteus*), containing lupinine and multiflorine (*Lupinus atlanticus*, *Lupinus palaestinus*, *Lupinus anatolicus*, *Lupinus digitatus*, *Lupinus pilosus*, and *Lupinus cosentinii*), and containing multiflorine (*Lupinus micranthus*). Within a given group, certain species can be, in most cases, further distinguished by the presence of other major alkaloids. The discrimination of species based on the total alkaloid content was found to be less reliable because of the significant intra-species variations, as well as the influences of environmental factors on the seed alkaloid content.

## 1. Introduction

The genus *Lupinus* consists of two geographically isolated groups of species: the Old World lupins (OWL) and the New World lupins [1]. A majority of the species within the genus belong to the New World group, which is distributed in both North and South America [2,3]. In the Mediterranean Basin and North Africa, 12 OWL species have been described, including three lupin crops, *Lupinus albus* L., *Lupinus angustifolius* L., and *Lupinus luteus* L. [4]. Subsequently, a Latin diagnosis of three new OWL species, *Lupinus anatolicus* Swiec. et Swiec. [5], the interspecific hybrid *Lupinus* × *hispanicoluteus* Swiec. et Swiec. [6], and *Lupinus mariae-josephi* H. Pascual [7], was presented.

Two sections related to seed coat texture have been distinguished among the OWL: the smooth-seeded species (section *Malacospermae*: *L. albus* L., *L. angustifolius* L., *L.* × *hispanicoluteus* Swiec. et Swiec, *Lupinus hispanicus* Boiss. & Reut., *L. luteus* L., and *Lupinus micranthus* Guss.) and the rough-seeded species (section *Scabrispermae: Lupinus atlanticus* Glads., *Lupinus cosentinii* Guss., *Lupinus digitatus* Forsk., *Lupinus palaestinus* Boiss., *Lupinus pilosus* Murr., *Lupinus princei* Harms, and *Lupinus somaliensis* Baker) [4]. The rough-seeded lupins are similar in terms of their morphological, chemical, and cytological features, and their genetic relationships were confirmed by successful crosses [2,4,8,9,10,11]. Despite their variable genome sizes and chromosomal numbers, the rough-seeded lupins form a strongly supported monophyletic group in phylogenetic analyses [2,12]. The smooth-seeded lupins form a heterogeneous group of species separated by major genetic barriers [1,13]. Two OWL species do not fit into this division scheme. *L. anatolicus* Swiec. et Swiec was initially characterized as a smooth-seeded species [5,14], but, based on further studies, was assigned to the *Scabrispermae* section [2]. *L. mariae-josephi* H. Pascual forms a distinct line in the phylogenetic analysis, with the seed coat texture being intermediate between that of the smooth- and rough-seeded lupins [15].

Lupins used in farming systems provide undeniable benefits related to their ability of symbiotic nitrogen fixation, their rotational benefits to succeeding crops, as well as their adaptation to poor soils and lower demands on the soil when compared with other crop species [16]. Nevertheless, the commercial values of lupins are primarily based on their production of seeds, which are used as fodder and food, being a good source of protein, dietary fiber, and oil [16]. One of the antinutritional factors of lupins is their alkaloids, which are synthesized in the aerial parts of the plants and accumulate in their seeds [17]. Lupin alkaloids are important secondary metabolites that play roles as defense or signal compounds, but at the same time, show neurotoxic effects on humans and animals [18]. Consequently, a limit of 0.02% of the seed dry weight has been assessed as a safe alkaloid content when used for food and feeding purposes [19]. Owing to the intensive breeding efforts, the seed alkaloid contents of modern lupin cultivars are often clearly lower than the accepted industry threshold [20,21].

**Figure 1:**
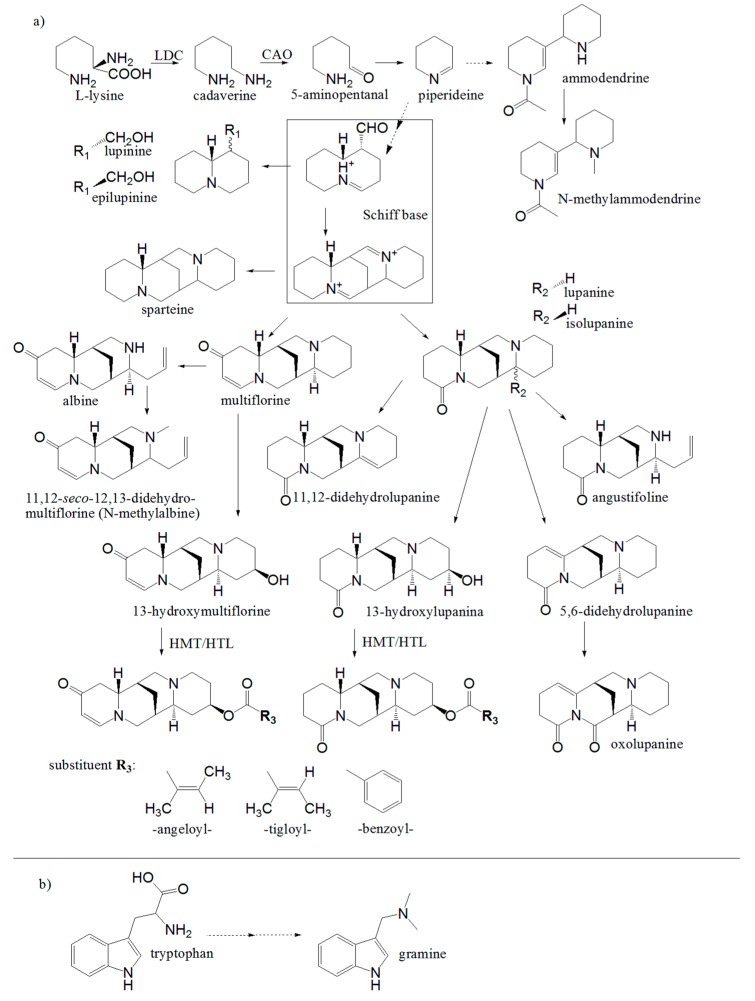
Putative biosynthetic pathway of alkaloids in *Lupinus* plants; (**a**) Lysine-derived: quinolizidine alkaloids (QAs), partly published by Bunsupa et al. (2012b) and Aniszewski (2007), and piperidine alkaloids (e.g., ammodendrine); (**b**) Tryptophan-derived indole alkaloid gramine; *LDC*-lysine decarboxylase [27]; *CAO*-copper amine oxidase [30], *HMT/HLT*-(−)-13α-hydroxymultiflorine/(+)-13α-hydroxylupanine O-tigloyltransferase [26]. Dotted lines denote uncharacterized or multistep reactions.

Plants of the genus *Lupinus* contain lysine-derived quinolizidine alkaloids (QAs) (Figure 1A), but they can also contain bipiperidine alkaloids (e.g., ammodendrine) [22] (Figure 1A), and tryptophan-derived, simple indole alkaloids (e.g., gramine) [23] (Figure 1B). Most lupin species possess particular alkaloid profiles in which major alkaloids (relative abundance > 1% of the total alkaloid content) and minor alkaloids (relative abundance < 1% of the total alkaloid content) can be distinguished, providing so-called alkaloid fingerprints [18,22].

The alkaloid biosynthetic pathway of the lupins has not yet been fully characterized [17] (Figure 1). The best-studied pathway at the genetic level is QA biosynthesis in narrow-leafed lupin and other crop species [24,25,26,27,28,29,30,31,32,33]. For further lupin breeding, which includes the domestication of new species, it is important to unravel alkaloid biosynthesis and to valorize lupin genetic resources [34,35,36]. The total contents and the relative abundances of individual QAs in the seeds of *L. angustifolius* (329 accessions) and *L. albus* (367 accessions) collected in the Polish *Lupinus* Genebank were explored, with consideration for the classes of origin, in our previous reports [20,21]. The aim of the present study was to explore the alkaloid profiles in the seeds of the remaining OWL species. The chromatographic fingerprints of the OWL alkaloids were evaluated based on their potential use in species discrimination.

## 2. Results

Alkaloid profiles for the seeds of 10 OWL species, encompassing 73 accessions, were investigated. The detailed results of the total contents and relative abundances of individual alkaloids in the assessed species are presented in Table S1, while the mean values of the major alkaloids (relative abundance > 1% of the total alkaloid content, in at least one individual per species) for each OWL are summarized in Table 1. The results obtained for the three lupin crops (*L. angustifolius*, *L. albus*, and *L. luteus*) were included in both overviews for comparative purposes. In the 13 OWL species, 20 alkaloids were detected, with 19 being major alkaloids (Table 1, Table S1).

### 2.1. OWL Qualitative Alkaloid Composition

For the individual species, 2–14 alkaloids were identified (excluding residual alkaloids of relative abundance < 0.1% of the total alkaloid content) (Table S1). When only major alkaloids were considered, two to nine alkaloids per species were detected (Table 1). In certain cases, however, particular major alkaloids were not detected at all, or their relative abundances were < 1% of the total alkaloids in some individuals within a species (Table 1, Table S1). Such situations, however, did not involve alkaloids having the greatest relative abundances within a species.

**Figure 2:**
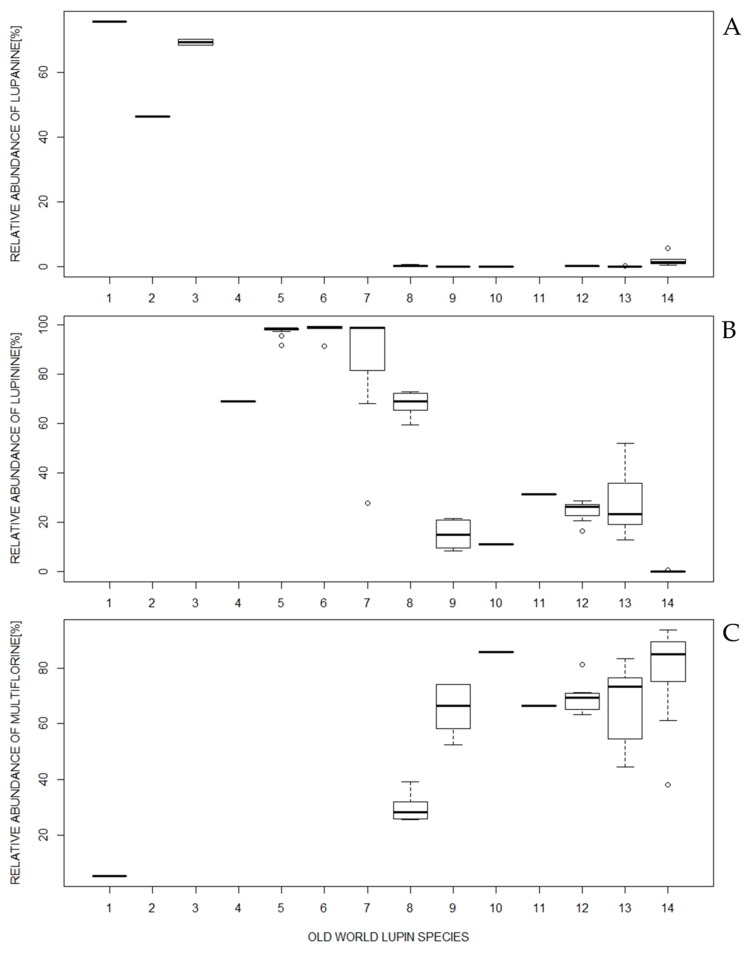
Boxplot showing relative abundances of the three most abundant alkaloids: (**A**) lupinine, (**B**) lupanine, and (**C**) multiflorine within 13 Old World lupin species (based on data presented in Table S1). The species are arranged in the following order: *Lupinus albus* (1), *L. angustifolius* (2), *L. mariae-josephi* (3), *L. luteus* (4), *L. hispanicus* subsp. *hispanicus* (5), *L. hispanicus* subsp. *bicolor* (6), *L.* × *hispanicoluteus* (7), *L. atlanticus* (8), *L. palaestinus* (9), *L. anatolicus* (10), *L. digitatus* (11), *L. cosentinii* (12), *L. pilosus* (13), and *L. micranthus* (14).

Lupinine was the most frequently occurring major alkaloid in the OWL (detected in 10 species and assessed as a major alkaloid in 9 species) (Figure 2, Table 1, Table S1). In contrast, in the remaining four species, lupinine could not be detected (*L. angustifolius*, *L. albus*, and *L. mariae-josephi*) or was assessed as a minor alkaloid (relative abundance < 1% of the total alkaloid content; *L. micranthus*). Therefore, two groups of species might be determined in OWL, those containing and those lacking lupinine as a major alkaloid. The group of nine species having lupinine as a characteristic alkaloid (Table 1) could be broadened to include *L. princei* because, according to Wink et al. [22], this species contains epilupinine, which is a lupinine epimer (Figure 1A) [17].

Among the four species in which lupinine was not a major alkaloid, *L. angustifolius*, *L. albus*, and *L. mariae-josephi* contained lupanine as their most abundant alkaloid, and *L. micranthus* contained multiflorine as its most abundant alkaloid (Table 1).

For alkaloids with the greatest relative abundances within a species, lupanine and multiflorine were the next most commonly occurring after lupinine (Table 1). Lupanine was present in nine species (Figure 2, Table S1), although its relative abundance exceeded 1% of the total alkaloid content in only four species (Table 1). Multiflorine was detected in eight species, and it was a major alkaloid in all of them (Figure 2, Table 1, Table S1).

Using the observed alkaloid ratios of the three most abundant alkaloids, lupinine, lupanine, and multiflorine, we distinguished four groups of OWL. Thus, specific alkaloid compositions could serve as the bases for further species differentiation within a group. Moreover, consistent relative abundance levels of major alkaloids were observed for seed samples obtained from different sources (genebanks/environments), which further strengthened the potential to discriminate species based on their qualitative alkaloid profiles. The four groups of OWLs are described in the subsections below (Section 2.1.1, Section 2.1.2, Section 2.1.3 and Section 2.1.4).

#### 2.1.1. Species Possessing Lupanine as the Most Abundant Major Alkaloid

Three OWL species included in this group were characterized by the lack of lupinine in addition to the outstanding amounts of lupanine (Figure 2A, Table 1, Table S1). Their further indicative features are as follows:

- *L. albus* and *L. angustifolius*, contained two other alkaloids in common, 13-hydroxylupanine and angustifoline. Both species could be distinguished using albine, ammodendrine, and multiflorine (and its derivatives), which were present in *L. albus* only, while *L. angustifolius* contained isolupanine as well as greater proportions of 13-hydroxylupanine and angustifoline.
- In *L. mariae-josephi*, lupanine and its derivatives were predominant. Albine and ammodendrine were not detected, while angustifoline and multiflorine were not major alkaloids for this species.

#### 2.1.2. Species Possessing Lupinine as the Most Abundant Major Alkaloid

Three OWL species classified into this group were also characterized by the lack of multiflorine and lupanine (Figure 2B, Table 1, Table S1). Individual species could be distinguished as follows:

- *L. luteus* contained sparteine as an additional, major alkaloid, and the sparteine content was greatest in this species compared with all the other OWLs. Moreover, ammodendrine is present.
- In both the subspecies of *L. hispanicus*, lupinine is predominant (~97%). *L. hispanicus* subsp. *hispanicus* was characterized as having gramine as the second major alkaloid, while for *L. hispanicus* subsp. *bicolor*, gramine was the major alkaloid in only one accession.
- *L. × hispanicoluteus* had a lupinine content that ranged from 28% to 99%, and gramine comprised 0.5–62% of the total alkaloid content. Moreover, sparteine, as well as ammodendrine and its derivatives, were also occasionally detected. In comparison, in the *L. luteus* parental lines (Wt96839, Wt98066, and Wt98067), lupinine (58–74%), sparteine (22–41%), ammodendrine (~0.5–2%), and sometimes gramine, were detected, while the *L. hispanicus* parental line (Wt96383) was distinguished by greater proportions of lupinine (>90%) and gramine as the major alkaloids. Because of recombined arrangements of parental alleles in each of the analyzed *L. × hispanicoluteus* accession, they may be considered as a separate genotype [6]. The resulting diverse alkaloid compositions make the identification of this hybrid species, although possible, more difficult than those of other OWLs.

#### 2.1.3. Species Possessing Lupinine and Multiflorine as the Two Major Alkaloids with the Greatest Relative Abundances

Six OWL species included in this group had different lupinine/multiflorine ratios (Figure 2B,C, Table 1, Table S1). Another common feature within the group was the lack of lupanine as a major alkaloid. The following factors were characteristic of individual species:

Species with a Greater Relative Abundance of Lupinine than Multiflorine.

- In *L. atlanticus*, the most distinguishing feature was the 2:1 ratio of the relative abundance of lupinine to multiflorine. In addition to the multiflorine derivatives detected in certain accessions, no other major alkaloids were detected within this species.

Species with a Greater Relative Abundance of Multiflorine than Lupinine.

- *L. palaestinus* was characterized as having the greatest proportions of ammodendrine and sparteine within the group. The relative abundance of ammodendrine was also the highest among the entire OWL group.
- *L. anatolicus* was unique, with its multiflorine content being the greatest among the group members and eight times greater than its lupinine content. Moreover, no additional major alkaloids were detected.
- *L. digitatus*, like *L. anatolicus*, did not have additional major alkaloids, but the ratio of lupinine to multiflorine was close to 1:2.
- The *L. cosentinii* and *L. pilosus* alkaloid profiles were considerably similar, with lupinine to multiflorine ratios of ~1:2. *L. cosentinii* could be distinguished by the lack of sparteine, while this alkaloid was present in certain *L. pilosus* accessions.

#### 2.1.4. Species Possessing Multiflorine as the Most Abundant Major Alkaloid

- *L. micranthus* has a distinct alkaloid composition, with dominating relative abundances of multiflorine and its derivatives (Figure 2C, Table 1, Table S1). Lupanine and angustifoline were also detected in all the accessions, although, in certain cases, they did not exceed 1% of the total alkaloid content. Nonetheless, the relative abundance of angustifoline in *L. micranthus* was the second greatest compared with the other OWLs. Additionally, in particular accessions, albine could be detected. Other than in this species, it was only found in *L. albus*.

**Figure 3:**
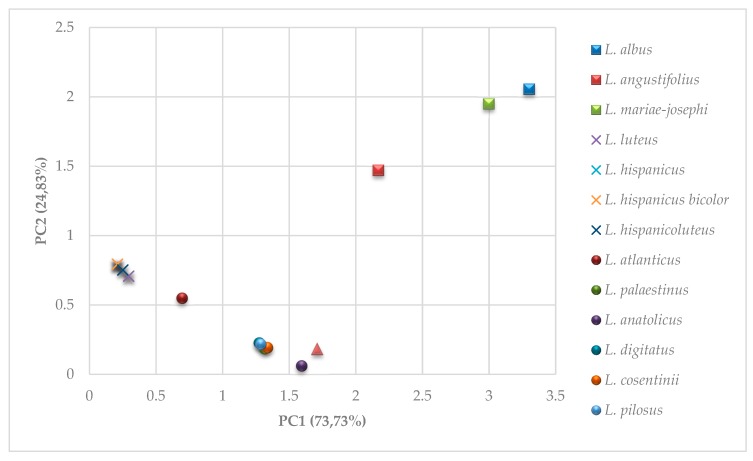
Principal component analysis (PCA) of 13 Old World lupin species (including two *L. hispanicus* subspecies) characterized by the three most abundant alkaloids (lupanine, lupinine, and multiflorine). PC1 and PC2 components explained a combined 98.56% of the variance. Original data were expressed in relative abundance (%) before the PCA.

A principal component analysis (PCA) carried out for 13 OWL species (including two *L. hispanicus* subspecies) with regards to the relative abundances of 20 alkaloids detected showed distinctions among three species, *L. albus*, *L. angustifolius,* and *L. mariae-josephi*, while the remaining species clustered together (Figure S1). To better reveal the relationships between the OWL species and to increase graphic resolution, in the two subsequent rounds of PCA the dataset was restricted to the six most distinguishing alkaloids (lupanine, lupinine, multiflorine, sparteine, gramine, and 13-angelolyoxomultiflorine) (Figure S2), and the most abundant alkaloids (lupinine, lupanine, and multiflorine) (Figure 3), respectively. These results showed again that the qualitative alkaloids compositions of *L. albus*, *L. angustifolius*, and *L. mariae-josephi* were clearly different than those of the remaining species. Moreover, the clustering of the species with the highest relative abundance of lupinine and multiflorine was also observed, as presented in Section 2.1.2, Section 2.1.3 and Section 2.1.4 (Figure 3, Figure S2).

### 2.2. OWL Total Alkaloid Contents

#### 2.2.1. Intraspecies Variation

A statistical comparison of the intraspecies variability among total seed alkaloid contents could be assessed for six species (including two *L. hispanicus* subspecies) that had at least four available accessions (Table 2). Among them, statistical differences (at the 0.01 significance level) were observed in all the species, except *L. cosentinii* and *L. hispanicus* subsp. *bicolor*. Moreover, significant differences in the total alkaloid contents of their seeds were also observed when comparing the same species retrieved from different genebanks (environments) (Tables S2 and S3). For the majority of the compared species, the environment had significant, varied impacts on the total alkaloid content of their seeds.

#### 2.2.2. Attempts of Interspecies Analyses

An analysis of variance applied to nine species collected from the Polish *Lupinus* Genebank showed significant differences among species based on their total seed alkaloid contents (F_calc._ = 53.75 > F_0.01_ = 2.63). To further examine the differences among species, their division into homogenous groups was tested. The investigated OWLs could be statistically divided into three homogenous groups (1, high total alkaloid content; 2, medium content; and 3, low content) (Table 3). *L. mariae-josephi* was distinct from the other species involved in the analysis, having the greatest alkaloid content and being the only member of the first group. The second and the third alkaloid groups consisted of species classically categorized as smooth- and rough-seeded lupins, respectively. No clear correlations between homogenous groups and their representatives’ origins or occurrences, as described by Gladstones [4], were observed.

## 3. Discussion

Lupin alkaloid profiles have been previously examined; however, OWL species were either not the main research focus, the survey was dedicated to a limited number of OWL species, or it was restricted to one accession per species [5,22,37,38,39,40,41,42]. Here, we presented a joint evaluation of the alkaloid profiles in the seeds of 13 OWL species, encompassing the recently discovered *L. anatolicus*, *L. mariae-josephi*, and *L. × hispanicoluteus*, and in most cases, incorporating several accessions within the species. The aim of our research was to provide a comprehensive assessment of similarities and differences on the basis of alkaloid fingerprints of all OWL species, and with particular attention not only to alkaloid occurrences but also the proportions of the relative abundances of the individual alkaloids. Our study updates and expands the previous analysis of Wink et al. [22], which covered 11 OWL species (including the no longer available *L. princei*), represented by one accession per species. In the 13 analyzed OWL species, we detected 20 alkaloids, and identified 19 as being major alkaloids (Table 1, Table S1), while previously, 14 major alkaloids had been identified in the OWLs [22].

The summary of the major alkaloids detected in the 13 OWLs (Table 1) provides the qualitative alkaloid composition of a given species, showing the distinctive alkaloids that each might possess. The analysis of the qualitative alkaloid compositions among the OWL species showed that certain subgroups might be distinguished based on common patterns among the major alkaloid profiles. Furthermore, in many cases, individual major alkaloids might serve as additional factors of species discrimination within these subgroups. Consistent results for the relative abundances of major alkaloids were also achieved for seed samples of the same OWL species retrieved from different genebanks (environments), which confirmed the potential ability to discriminate among species based on their seeds’ qualitative alkaloid profiles. From the perspective of the seed coat texture categories, we observed that the rough-seeded species formed a more homogenous group, while the smooth-seeded lupins had considerably diverse qualitative alkaloid compositions. It is noteworthy that for three species, only a limited number of their representatives have been analyzed (*L. digitatus—*one accession, *L. anatolicus*—one accession, *L. mariae-josephi*—2 two accessions) and additional accessions would strengthen the final conclusions. No additional genetic resources are at present available worldwide for these OWL species however, our results on alkaloid patterns are in line with those reported by other authors [5,14,38,42]. The closer relationships among rough-seeded species are in accordance with the findings of previous analyses based on seed globulin profiles, isozymes, and phylogenies incorporating internal transcribed spacer sequences [2,9,11]. Such differences might be triggered by adaptations to environmental changes in the Mediterranean region and northern Africa during evolution [2].

An overview of the OWL subgroups that was distinguished based on quantitative alkaloid profiles in seeds versus putative lupin alkaloid biosynthetic pathways (Figure 1, Table 1) showed that the key step is the transformation of the Schiff’s base, which crucially impacted the major alkaloids created. This process might give rise to bicyclic lupinine, tetracyclic lupanine, multiflorine, and sparteine, or combinations. Other QAs appear to be formed by further conversions at later stages, except piperidine and indole alkaloids, which are created separately. The relative abundance levels of three major alkaloids, lupinine, lupanine, and multiflorine, were useful in the discrimination of OWL subgroups having similar alkaloid patterns. Relative proportions of these alkaloids differed among species, but remarkably when lupinine was a major alkaloid, lupanine was not. At the same time, parallel occurrences of lupinine and multiflorine were observed in some species, while in *L. micranthus* multiflorine predominated. The conversion leading to sparteine formation was less prevalent in OWLs, with sparteine being a major alkaloid in only a few species. In further investigations aimed at unraveling lupin alkaloid biosynthesis, understanding the Schiff’s base general background, as well as the fundamentals of its conversion into the four, mentioned major alkaloids, will be important.

Because the seed alkaloid content, beyond the genetic background, is strongly influenced by environmental factors, such as drought, temperature, fertilization, and/or soil pH [43,44,45,46], the statistical analyses concerning the total alkaloid contents were carried out using accessions gathered in the Polish *Lupinus* Genebank that originated from one field experiment. However, this collection did not include the rough-seeded *L. digitatus* or two no longer available species, *L. princei* and *L. somaliensis*. The observed significant intra-species variation of the quantitative alkaloid content revealed that the alkaloid content is not a fixed value within a species and, therefore, may not be a reliable parameter for species discrimination (Table 2). This was further confirmed by comparing results obtained for a single species from different environments, which demonstrated the significant influence of environmental factors on the total alkaloid content (Table S3). Large interindividual variability in the seed alkaloid content was also observed in previously investigated lupin crop species [20,21].

Regardless of the intra-species variation, we investigated the variation among the species and the possibility of dividing them into homogenous groups based on their total alkaloid contents (Table 3). This analysis revealed the distinctiveness of *L. mariae-josephi*, which was different from the other OWL species, having the greatest alkaloid content and being the only member of the first homogenous group. This species was previously identified as a diverse line within the OWL phylogeny, without a clear affinity to either the smooth- or rough-seeded group [15]. In the second and third homogenous groups, we further observed statistical differences among species classically categorized as smooth- and rough-seeded lupins, respectively. The group characterized by the lowest alkaloid content consisted of the wild, rough-seeded species. In contrast, *L. hispanicus*, which belongs to the secondary gene pool of cultivated *L. luteus*, and *L. × hispanicoluteus* (*L. hispanicus* × *L. luteus* hybrid) [6,47], were assigned to the medium alkaloid content group. The observed division based on the total alkaloid content was more or less in line with OWL classifications based on seed coat texture. These results, together with the greater total alkaloid content in the smooth-seeded lupins over the rough-seeded lupins, were rather surprising and have not been reported previously. From an evolutionary point of view, wild accessions would be expected to exhibit primitive features, such as a rough seed coat and a high alkaloid content. Interestingly, similar results were found in *Pisum*, in which primitive taxa had the lowest oligosaccharide contents [48]. Because certain species were not present or were represented by single accessions, these results need further support. However, the major constraint is again the limited genetic resources for wild OWL species available worldwide.

Several other observations based on the presented OWLs qualitative and quantitative alkaloid patterns are noteworthy. The interspecies analysis based on the total alkaloid content showed that *L. anatolicus* [5] was not statistically different from *L. pilosus* or *L. palaestinus* (Table 3), but differed from *L. micranthus*, to which it was initially considered phenotypically similar [5]. These results corroborated those of Aïnouche and Bayer [37] and Aïnouche et al. [2], which described *L. anatolicus* as being related to the *Pilosus-Palaestinus* subclade of *Scabrispermae*. Nonetheless, PCA results showed that the distance of *L. anatolicus* to four rough-seeded species, *L. pilosus*, *L. palaestinus*, *L. cosentinii,* and *L. digitatus*, is more or less the same as to *L. micranthus* (Figure 3, Figure S2). *L. micranthus* was found to exhibit a distinctive alkaloid profile compared with those of other OWLs having multiflorine and its derivatives, as well as albine and angustifoline, as the most abundant seed alkaloids. A similar pattern of seed alkaloids has also been reported by Muzquiz et al. [42]. The distinctiveness of *L. micranthus* was also observed based on its position in the phylogenetic tree [12], seed proteomic data [49], nuclear DNA content [8] and karyotype analysis [50], as well flavonoid [51] and isozyme contents [11], and consequently, an intermediate position between smooth- and rough-seeded lupins has been proposed. The presented PCA (Figure 3, Figure S2) indicated that *L. micranthus*’ qualitative alkaloid content was considerably different than those of other smooth-seeded lupins and more similar to rough-seeded species.

Legumes’ secondary metabolites, including quinolizidine alkaloids, have been previously assessed as chemotaxonomic markers [12,52]. When the presence/absence of individual quinolizidine alkaloids has been investigated within the *Lupinus* genus, their systematic values were limited. For example, bicyclic lupinine could be detected in the rough-seeded lupins, certain smooth-seeded species (i.e., *L. luteus*) and some North and South American species [12,22]. Similarly, a comparison of molecular phylogeny with the occurrences of quinolizidine alkaloids within the Papilionoideae subfamily showed that, in some cases, not all members of a monophyletic taxon share a chemical characteristic (i.e., tribe Crotalaria had pyrrolizidine instead of quinolizidine alkaloids) [12]. Nonetheless, it is undeniable that phytochemical studies significantly contribute to understanding the diversity in plants, by providing useful information regarding similarities and differences between and within species. The groups distinguished within the current investigation based on seed alkaloid profiles at least partially reflect currently accepted phylogenetic relationships among OWLs [2,12] and could be a useful supplemental tool in OWL species discrimination.

## 4. Materials and Methods

### 4.1. Plant Material

In total, 10 OWL species, *L. atlanticus* (11 accessions), *L. anatolicus* (1 accession), *L. cosentinii* (8 accessions), *L. hispanicus* subsp. *hispanicus* (13 accessions), and *L. hispanicus* subsp. *bicolor* (5 accessions), *L. micranthus* (8 accessions), *L. palaestinus* (6 accessions), *L. pilosus* (11 accessions), *L. digitatus* (1 accession), *L. mariae-josephi* (2 accessions), and the interspecific hybrid *L. × hispanicoluteus* (7 accessions) were considered in the analyses. The *L. × hispanicoluteus* parental lines, *L. hispanicus* subsp. *hispanicus* (Wt96383) and *L. luteus* (high alkaloid Wt96839 and low alkaloids Wt98066 and Wt98067), were investigated for comparative purposes (Table S1).

The seeds were retrieved mainly from the Polish *Lupinus* Genebank collection (Wiatrowo); however, for the species represented by limited resources, additional records were requested from the following other sources: The United States Department of Agriculture’s National Plant Germplasm System (Pullman, WA, USA), Genebank of the Leibniz Institute of Plant Genetics and Crop Plant Research (Gatersleben, Germany), and Instituto Nacional de Investigacion y Tecnologia Agraria y Alimentaria (Madrid, Spain). As precise information on the accessions’ collection sites are rarely specified in such databases, the geographical distribution covered by the incorporated materials was difficult to determine. Additional records were therefore gathered to obtain a total of at least five accessions per species (where possible). *L. anatolicus* and *L. digitatus* were each represented by one accession only in the world’s *Lupinus* germplasm resources, while *L. mariae-josephi* was represented by only two accessions. Two other species, *L. princei* and *L. somaliensis*, appeared to be no longer accessible in the *Lupinus* germplasm resources. The detailed list of OWL seed samples incorporated in this study, including their source(s), is presented as Table S2. The alkaloid profiles for the 10 species (73 accessions) considered in this study were combined with those of the three lupin crops, resulting in 13 evaluated OWLs. For *L. angustifolius* and *L. albus*, the average values of the total contents and the relative abundances of individual alkaloids were calculated from the results of earlier works [20,21]. For *L. luteus*, the average values of 20 representative accessions were estimated, because the alkaloid profiles of the entire Polish collection are not yet available.

The seeds of the accessions originating from the Polish *Lupinus* Genebank collection were grown in a field experiment in Wiatrowo, Poland, using a completely randomized design with two replicates (plot size of 1 m^2^ with 60 seeds per plot) in 2017. The seed samples were harvested after full maturity, and the representative samples used for further analyses contained 10 g of the milled seeds.

### 4.2. Extraction Method and Assessments of the Total Contents and Relative Abundances of Alkaloids in OWL Seeds

The seed samples were ground to a fine meal and dried overnight at 65 °C. For each accession, two technical replications, 0.5 g each, were prepared. To each 0.5 g sample, 5 mL of 5% trichloroacetic acid was added, and the sample was homogenized at room temperature for 15 min, followed by centrifugation at 5,600 × g for 10 min at 5 °C. The supernatant was decanted, and the above homogenization and centrifugation steps were repeated twice. A 15 mL aliquot of the supernatant was subsequently alkalized with 1 mL of 10 M sodium hydroxide and extracted three times with 15 mL of dichloromethane. The organic extracts were dried over anhydrous sodium sulfate and decanted into a new flask. Next, 20 µL of the internal standard (1 mg/mL caffeine solution) was added to the organic extract, and the solvent was vacuum dried. The residues were reconstituted in 300 µL of dichloromethane. The characterization of alkaloids was carried out using gas chromatography, GC-2014 (Shimadzu, Kyoto, Japan) operating with an FID detector with a sensitivity of 2 mV at 300 °C. A ZB-5 silica capillary column (30 m × 0.25 mm ID × 0.25 µm; Phenomenex, Torrance, CA, USA) was used. The injector temperature was 250 °C with the normal injection mode. The initial oven temperature of 180 °C was maintained for 2 min. The temperature was then increased by 5 °C/min up to 300 °C and held for 10 min at 300 °C. The flow rate of the carrier gas helium was 1 mL/min, with a split ratio of 1:20. Alkaloid identification was performed by comparing the retention times of alkaloid standards obtained from the Institute of Bioorganic Chemistry, Polish Academy of Sciences, Poznan, Poland. Representative chromatograms for 13 OWL species were presented as Figure S3. A quantitative analysis was carried out using a linear calibration curve made for lupanine using caffeine as internal standard (calibration equation y = 0.0092x – 0.0043, R^2^ = 1; where x = the ratio of peak areas of all alkaloids detected vs. peak area of caffeine and y = alkaloid content [mg]). The total alkaloid content was assessed as the percentage of the sum of the alkaloids expressed in the seed dry weight (% of seed dry weight). The relative abundance of an alkaloid was assessed as the percentage of the total alkaloid content (sum of all alkaloids = 100%). Subsequently, the major alkaloids were estimated for each species (relative abundance > 1% of the total alkaloid content, detected in at least one accession). The reproducibility of the methods used for alkaloid extraction and measurement was tested by comparing the results obtained for the same seed samples that were analyzed in two different laboratories. Moreover, the stability of the results was periodically monitored by repeated analyses of already characterized samples over specific time intervals.

### 4.3. Analyses of OWL Species Variability in Terms of Seed Alkaloid Content and Composition

All 13 OWL species, encompassing 73 accessions, were incorporated into the analyses of the qualitative alkaloid profiles. Statistical analyses of the results related to the total alkaloid content in seeds were conducted. Owing to the influence of environmental factors on lupin alkaloid contents, only accessions derived from the Polish *Lupinus* Genebank collection (10 OWL species, 49 accessions) as originating from one filed experiment, were included in the statistical analyses (Table S1). The three lupin crops were not included in the statistical analyses because their alkaloid contents were analyzed in other experiments [20,21].

### 4.4. Statistical Analyses

For species having relatively large numbers of analyzed accessions (4–13), the statistical characteristics (measures of location and dispersion) were additionally calculated, and analyses of variance (F-statistics values) were performed to investigate differences between accessions within individual species [53]. The analyses of variance in the completely randomized design with unequal numbers of replications were also used to investigate the substantial differences across the same species derived from different genebanks/environments [53]. To test the homogeneity of the groups contained in the sets of OWL species considered in the analysis of variance, the Gabriel procedure was used [54]. The grouping procedure was stopped when the within-groups’ sum of squares became lower than the critical value for the overall F-test in the analysis of variance. This resulted in three homogenous groups of species.

To graphically present the results of the seed alkaloids compositions of the 13 OWL species (including two *L. hispanicus* subspecies) three rounds of PCA were performed [53,55]. In the first round, data on 20 detected alkaloids were incorporated. In the second round, a set of six correlated and the most differentiating alkaloids were replaced with two new, uncorrelated variables. In the third round, data on the three most abundant alkaloids were used. In all cases, the transformation into two-dimensional space did not result in a significant loss of information (less than 10%).

## 5. Conclusions

This study provides the most complete and up-to-date overview of the alkaloid profiles observed in the seeds of lupin species originating from the Mediterranean Basin. Our findings increase the current phytochemical knowledge of OWL species by providing new insights into their diversity as well as constituting another step towards the valorization of OWL genetic resources. We also demonstrated that patterns in qualitative alkaloid composition-based profiles may serve as supplemental tools in species discrimination within OWLs.

## Figures and Tables

**Table 1:** Nineteen major alkaloids (relative abundance > 1% of the total alkaloid content) detected in the seeds of Old World lupins. Mean values per species are shown (for details see Additional file 1: Table S1). 13-Hydroxymultiflorine detected in certain accessions was not identified as a major alkaloid. The data were calculated in relation to lupanine standard).

Species	Origin ^##^	Lupinine	Epilupinine	Multiflorine	11.12-secodidehydromultiflorine	13-tigloyloxymultiflorine	13-angelolyoxomultiflorine	Lupanine	13-hydroxylupanine	Isolupanine	11.12-didehydrolupanine	5.6-didehydrolupanine	Oxolupanine	13-benzoyloxylupanine	Sparteine	Angustifoline	Albine	Ammodendrine	N-methylammodendrine	Gramine
*L. angustifolius*	W							46.40 ^#^	35.60	2.50 ^#^						15.50 ^#^				
*L. albus*	W			5.52 ^#^	1.74 ^#^			76.06	8.23 ^#^							2.07 ^#^	4.48 ^#^	1.04 ^#^		
*L. luteus*	W	67.46	0.33 ^#^												28.86			1.40 ^#^		1.77 ^#^
*L. anatolicus*	W	11.12		85.68																
*L. palaestinus*	W	15.07		65.36	1.48 ^#^	0.96 ^#^						0.79 ^#^		1.12 ^#^	6.22			7.22	0.59 ^#^	
*L. cosentinii*	W	25.35		66.64	3.30	1.15 ^#^				0.34 ^#^		0.76 ^#^		1.36 ^#^						
	G	23.51		73.94	1.04 ^#^					1.13 ^#^										
*L. atlanticus*	W	66.68		30.35	1.35 ^#^															
	G	72.12		26.03	0.76 ^#^															
	U	63.99		34.40																
*L. pilosus*	*W*	27.76		63.85	2.67	0.87 ^#^								0.94 ^#^	1.31 ^#^			0.77 ^#^		
	U	28.06		69.56	0.77 ^#^					0.78 ^#^								0.51 ^#^		
*L. digitatus*	U	31.50		66.43																
*L. micranthus*	W			91.61			3.20										1.77			
	G			81.70			11.18	2.15 ^#^								3.20 ^#^		0.35 ^#^		
*L.* × *hispanicoluteus*	W	83.72													3.68 ^#^			1.81 ^#^	0.43 ^#^	10.31 ^#^
*L. hispanicus* subsa. *bicolor*	W	97.18																		2.62 ^#^
	U	98.29																		
*L. hispanicus* subsp. *hispanicus*	W	97.51													0.15 ^#^					2.20
*L. mariae-josephi*	W							68.42	5.12	18.27	4.05		3.01							
	I							70.30	4.76	20.78	3.48									

^#^ in certain accessions this alkaloid was not detected at all or was detected at a level < 1% of the total alkaloid contents. ^##^ W, Polish *Lupinus* Genebank Wiatrowo; G, Leibniz Institute of Plant Genetics and Crop Plant Research, Gatersleben (Germany); U, USDA National Plant Germplasm System; I, Instituto Nacional de Investigacion y Tecnologia Agraria y Alimentaria (Spain).

**Table 2:** Statistical comparison of the Old World lupin intraspecies variability with regard to total alkaloid content in their seeds (expressed as the percentage of the seed dry weight (DW)).

Species	Number of Accessions	Total Alkaloid Content (% of the seed DW). Mean Values for Species		Variation Coefficient (%)	F-Statistic for Differences Between Accessions
		Minimum	Maximum		
*L. palaestinus*	6	0.0774	0.2308	35.48	23.266 **
*L. cosentinii*	5	0.1317	0.2509	27.90	3.259
L. atlanticus	5	0.1263	0.2640	26.33	56.128 **
*L. pilosus*	6	0.0872	0.4517	54.23	77.281 **
*L. × hispanicoluteus*	7	0.0087	2.1189	78.31	17.829 **
*L. hispanicus* subsp. *bicolor*	4	0.6912	1.5982	36.75	5.302
*L. hispanicus* subsp. *hispanicus*	13	0.7743	1.9834	30.20	3.213 *

* Significant at 0.05. ** Significant at 0.01.

**Table 3:** Old World lupin species divided into homogenous groups based on the total content of alkaloids accumulated in their seeds (expressed as a percentage of the seed dry weight).

Homogenous Groups	Mean Values of Total Alkaloid Contents		Sum of Squares	Species
	Group	Species		
1	5.6611			*L. mariae-josephi*
2	1.0431	1.2101	0.5706	*L. hispanicus* subsp. *hispanicus* *L. hispanicus* subsp*. bicolor* *L.* × *hispanicoluteus* *L. micranthus*
		1.0772		
		0.9868		
		0.8984		
3	0.1765	0.2588	0.0946	*L. pilosus* *L. atlanticus* *L. cosentinii* *L. palaestinus* *L. anatolicus*
		0.1827		
		0.1799		
		0.1436		
		0.1177		

## Supplementary Materials

The following are available online at https://www.mdpi.com/2223-7747/8/12/548/s1, Table S1: Detailed results of the total contents and qualitative compositions of alkaloids in the 13 Old World lupin species, Table S2: List of OWL seed samples, and their sources, used in this study, Table S3: Statistical comparison of differences between the means of the seeds’ total alkaloid content (percentage of the seed dry weight) for species derived from different genebanks (environments), Figure S1: Principal component analysis (PCA) of 13 Old World lupin species characterized by 20 alkaloids with PC1 and PC2 explaining 99.01% variance. Original data were expressed in relative abundance (%) before the PCA, Figure S2: Principal component analysis (PCA) of 13 Old World lupin species characterized by the six most differentiating alkaloids (lupanine, lupinine, multiflorine, sparteine, gramine, and 13-angelolyoxomultiflorine). PC1 and PC2 components explained a combined 90.16% of the variance. Original data were expressed in relative abundance (%) before the PCA, Figure S3: Representative chromatograms showing GC separation of the alkaloids from the Old World lupin species. Here the *x*-axis shows the retention time (min), while the *y*-axis represents signal intensity (pA).
