# Supplementary material for: Chromatographic Fingerprinting of the Old World Lupins Seed Alkaloids: A Supplemental Tool in Species Discrimination
Source: Plants (Basel). 2019 Nov 27;8(12):548. doi: 10.3390/plants8120548 (PMC6963311; doi:10.3390/plants8120548)
Supplement: Supplementary file 1 [file plants-08-00548-s001.zip › Supplemenary Table S3.docx]

Supplementary Table S3. Statistical comparison of differences between means of seed’ total alkaloid contents (percentage of the seed dry weight) for species derived from different genebanks (environments). Differences were calculated for species in columns (2) versus species in rows (1).

| 1 2 | | Wiatrowo | | | | Getersleben | | | USDA | | |
| --- | --- | --- | --- | --- | --- | --- | --- | --- | --- | --- | --- |
|  |  | *L. cosentinii* | *L. micranthus* | *L. hispanicus bicolor* | *L. pilosus* | *L. atlanticus* | *L. cosentinii* | *L. micranthus* | *L. atlanticus* | *L. hispanicus bicolor* | *L. pilosus* |
| Wiatrowo | *L. atlanticus* | -0.0027 | 0.7157** | 0.8945** | 0.0761 | 0.4937** | 0.4577** | 0.5738** | 0.4060** | 2.3680** | 0.9195** |
|  | *L. cosentinii* |  | 0.7184** | 0.8972** | 0.0788 | 0.4965** | 0.4604** | 0.5765** | 0.4088** | 2.3707** | 0.9222** |
|  | *L. micranthus* |  |  | 0.1788 | -0.6396** | -0.2220 | -0.2580 | -0.1419 | -0.3097 | 1.6523** | 0.2038 |
|  | *L. hispanicus bicolor* |  |  |  | -0.8184** | -0.4008** | -0.4368** | -0.3207** | -0.4885** | 1.4735** | 0.0250 |
|  | *L. pilosus* |  |  |  |  | 0.4176** | 0.3816** | 0.4977** | 0.3300* | 2.2919** | 0.8434** |
| Getersleben | *L. atlanticus* |  |  |  |  |  | -0.0360 | 0.0801 | -0.0877 | 1.8743** | 0.4257** |
|  | *L. cosentinii* |  |  |  |  |  |  | 0.1161 | -0.0516 | 1.9103** | 0.4618** |
|  | *L. micranthus* |  |  |  |  |  |  |  | -0.1677 | 1.7942** | 0.3457** |
| USDA | *L. atlanticus* |  |  |  |  |  |  |  |  | 1.9620** | 0.5134** |
|  | *L. hispanicus bicolor* |  |  |  |  |  |  |  |  |  | -1.4485** |

* Significant differences 0.05

** Significant differences 0.01
