# Supplementary material for: Chromatographic Fingerprinting of the Old World Lupins Seed Alkaloids: A Supplemental Tool in Species Discrimination
Source: Plants (Basel). 2019 Nov 27;8(12):548. doi: 10.3390/plants8120548 (PMC6963311; doi:10.3390/plants8120548)
Supplement: Supplementary file 1 [file plants-08-00548-s001.zip › Supplementary Figure S3.pdf]

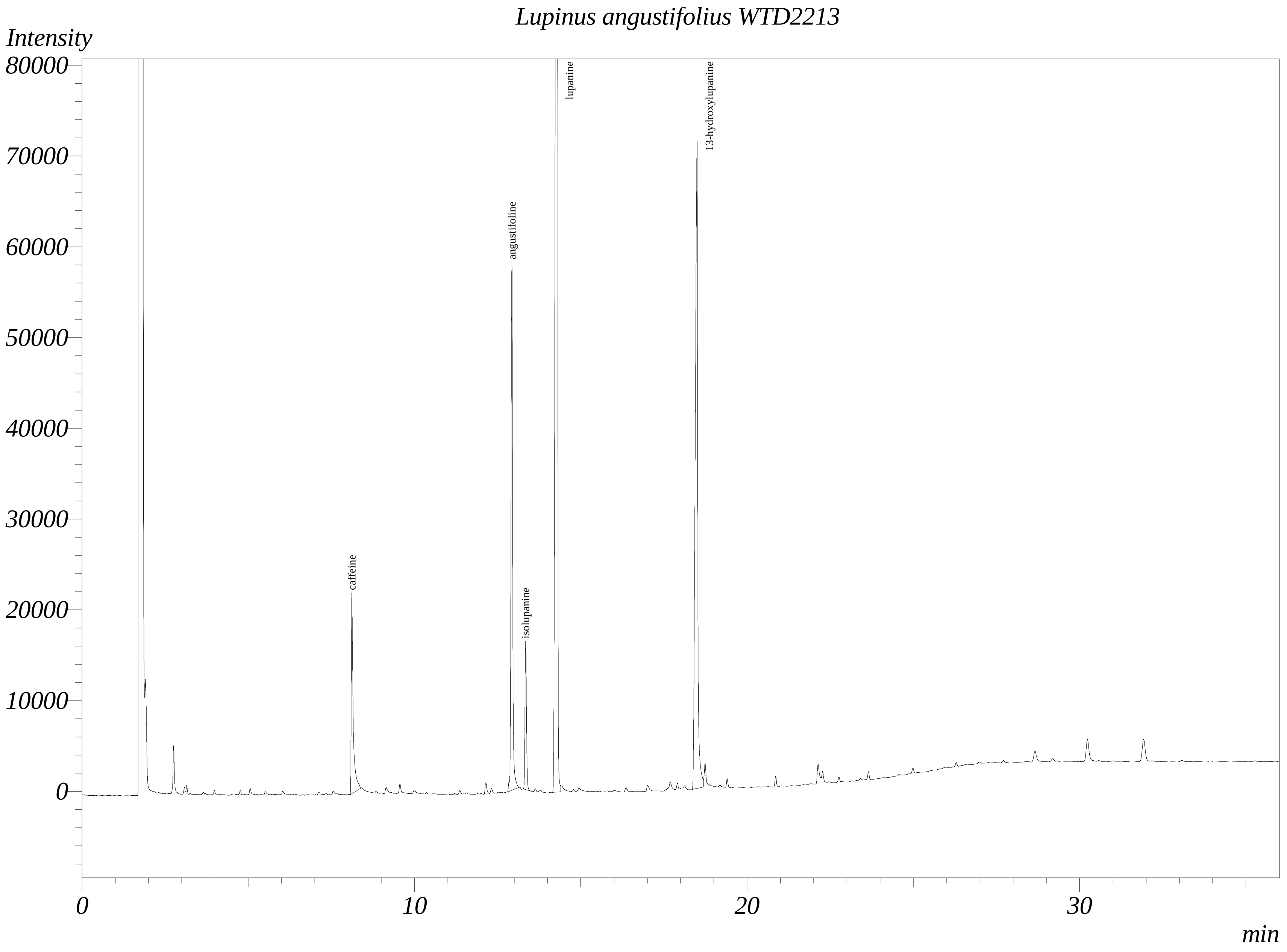
Lupinus angustifolius WTD2213
Intensity
80000
70000
60000
50000
40000
30000
20000
10000
0
0
10
20
30
min
caffeine
angustifoline
isolupanine
lupanine
13-hydroxylupanine

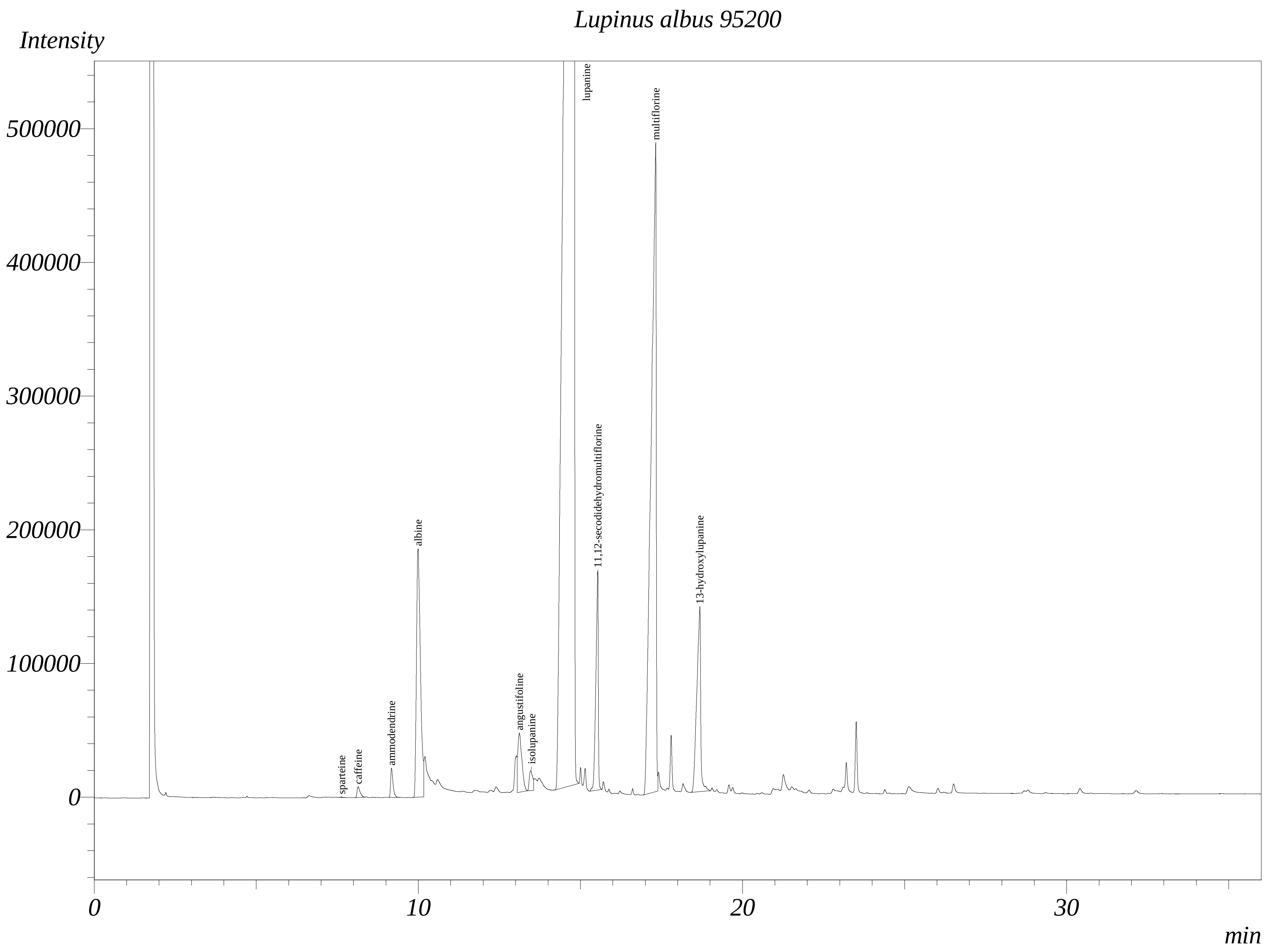

Lupinus albus 95200
Intensity
500000
400000
300000
200000
100000
0
sparteine
caffeine
ammodendrine
albine
angustifoline
isolupanine
lupanine
11,12-secodidehydromultiflorine
multiflorine
13-hydroxylupanine
0
10
20
30
min

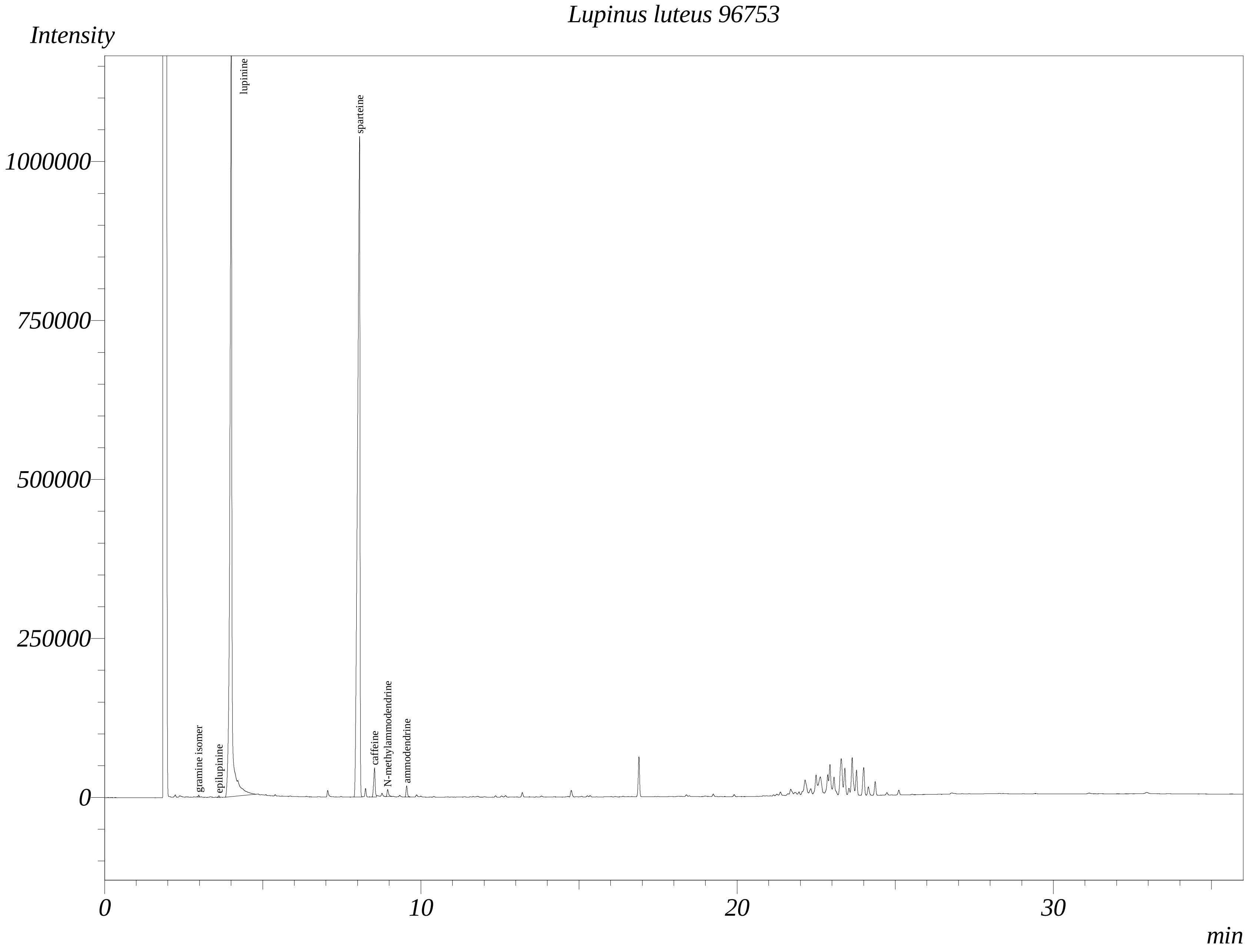
Lupinus luteus 96753
Intensity
1000000
750000
500000
250000
0
lupinine
sparteine
gramine isomer
epilupinine
caffeine
N-methylammodendrine
ammodendrine
0
10
20
30
min

Lupinus anatolicus 98698

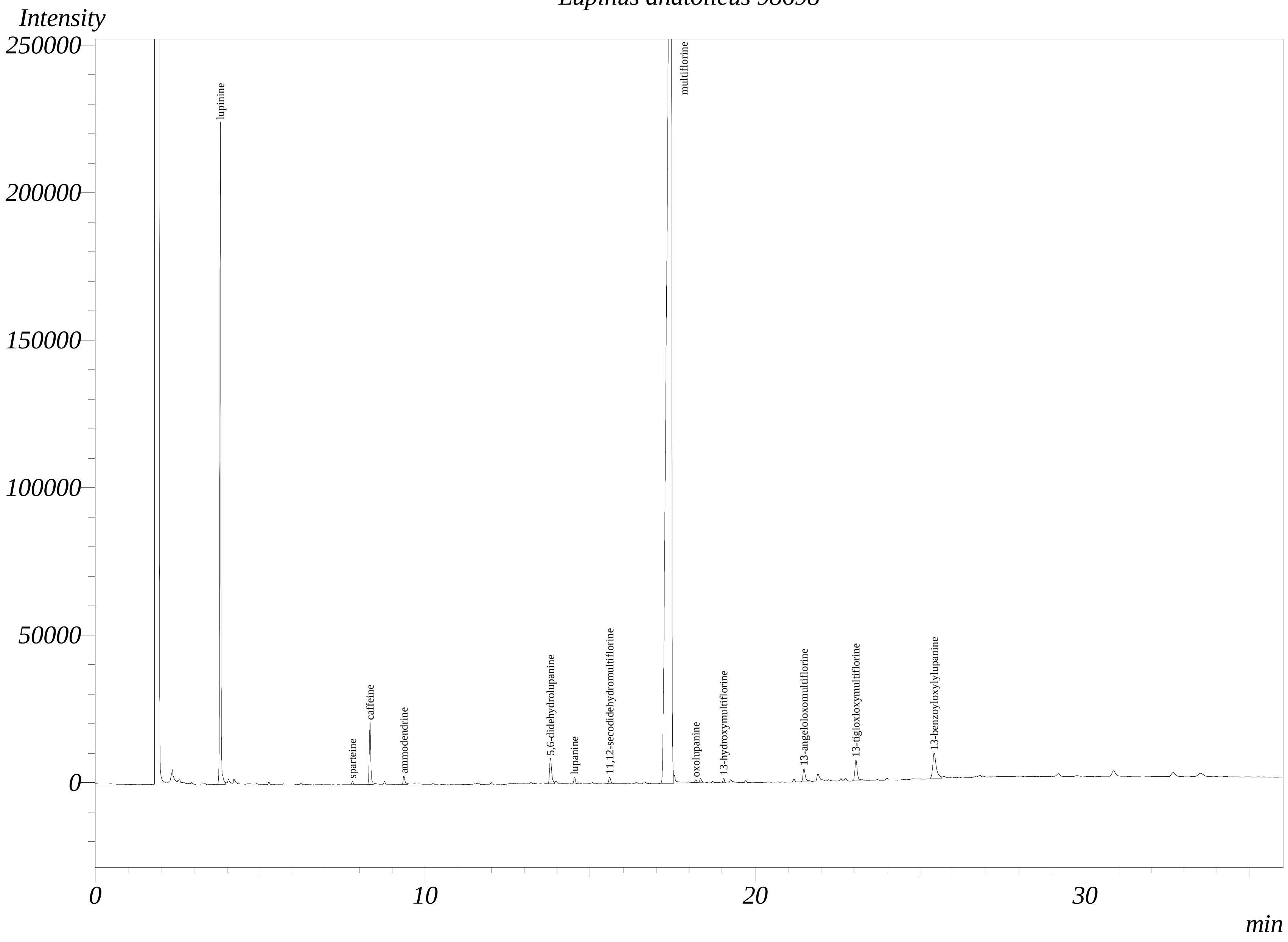

Lupinus palaestinus 98606

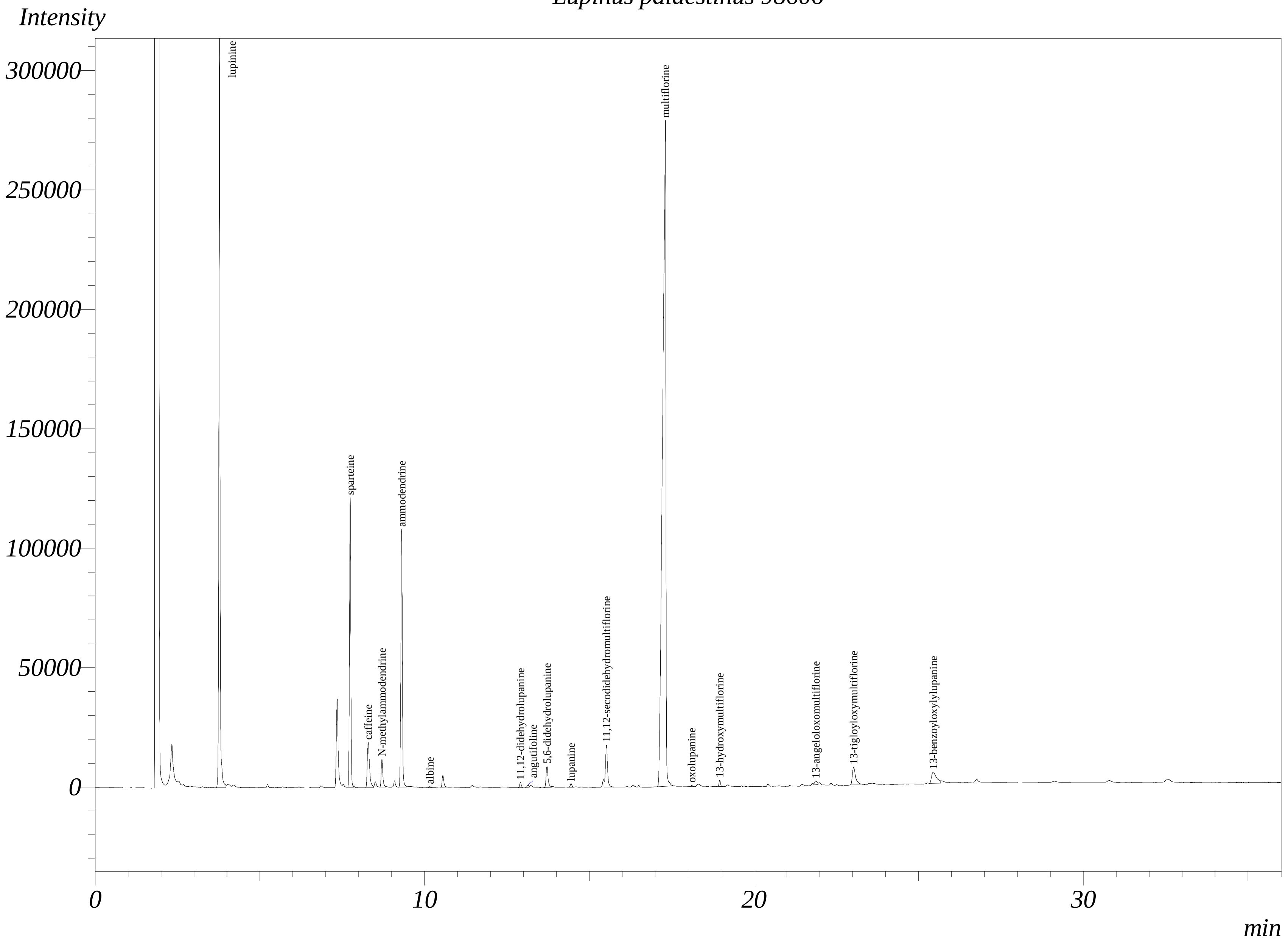

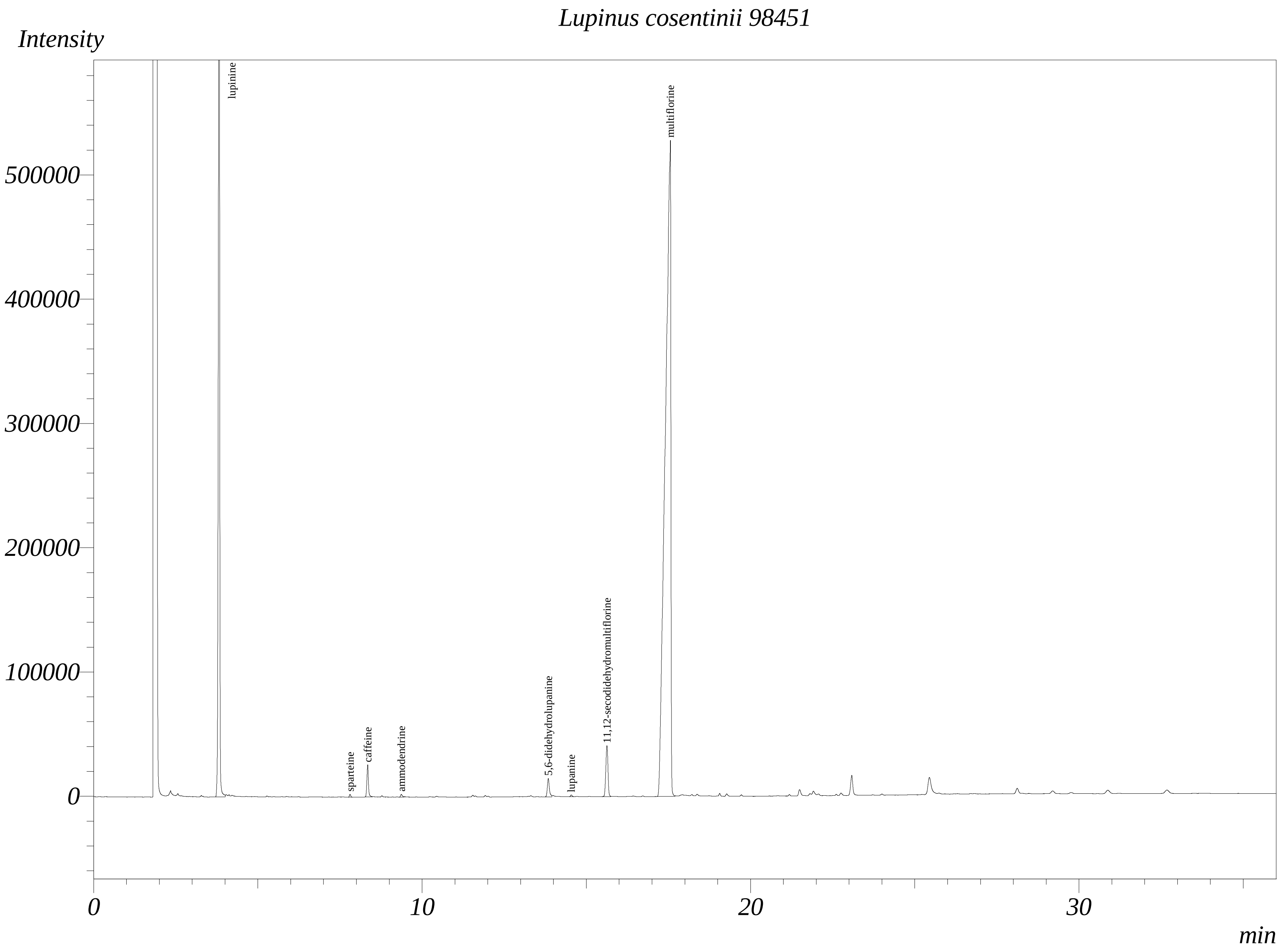

Lupinus cosentinii 98451
Intensity
500000
400000
300000
200000
100000
0
lupinine
sparteine
caffeine
ammodendrine
5,6-didehydrolupanine
lupanine
11,12-secodidehydromultiflorine
multiflorine
0
10
20
30
min

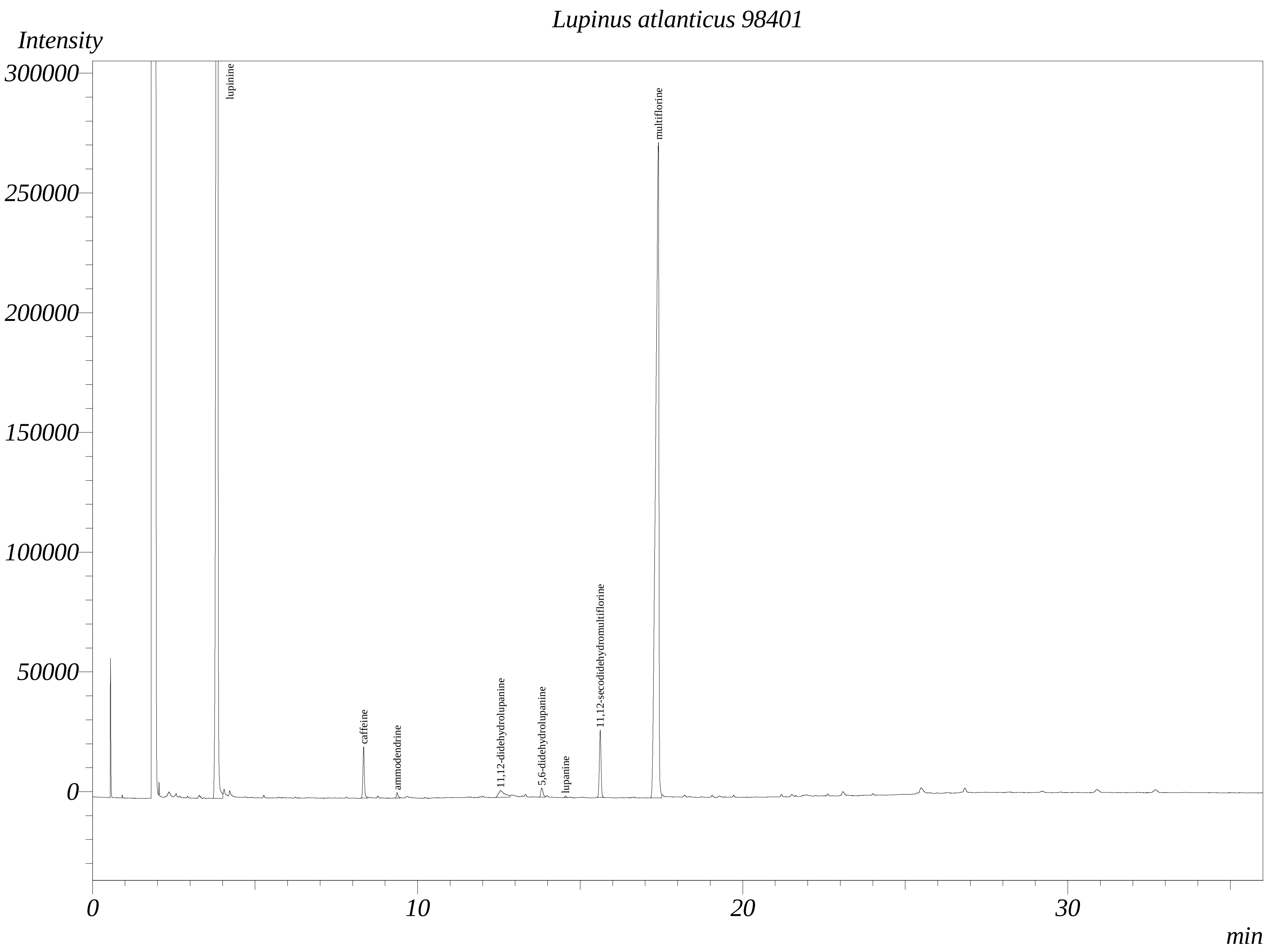

Lupinus atlanticus 98401
Intensity
300000
250000
200000
150000
100000
50000
0
0
10
20
30
min
lupinine
multiflorine
caffeine
ammodendrine
11,12-didehydrolupanine
5,6-didehydrolupanine
lupanine
11,12-secodidehydromultiflorine

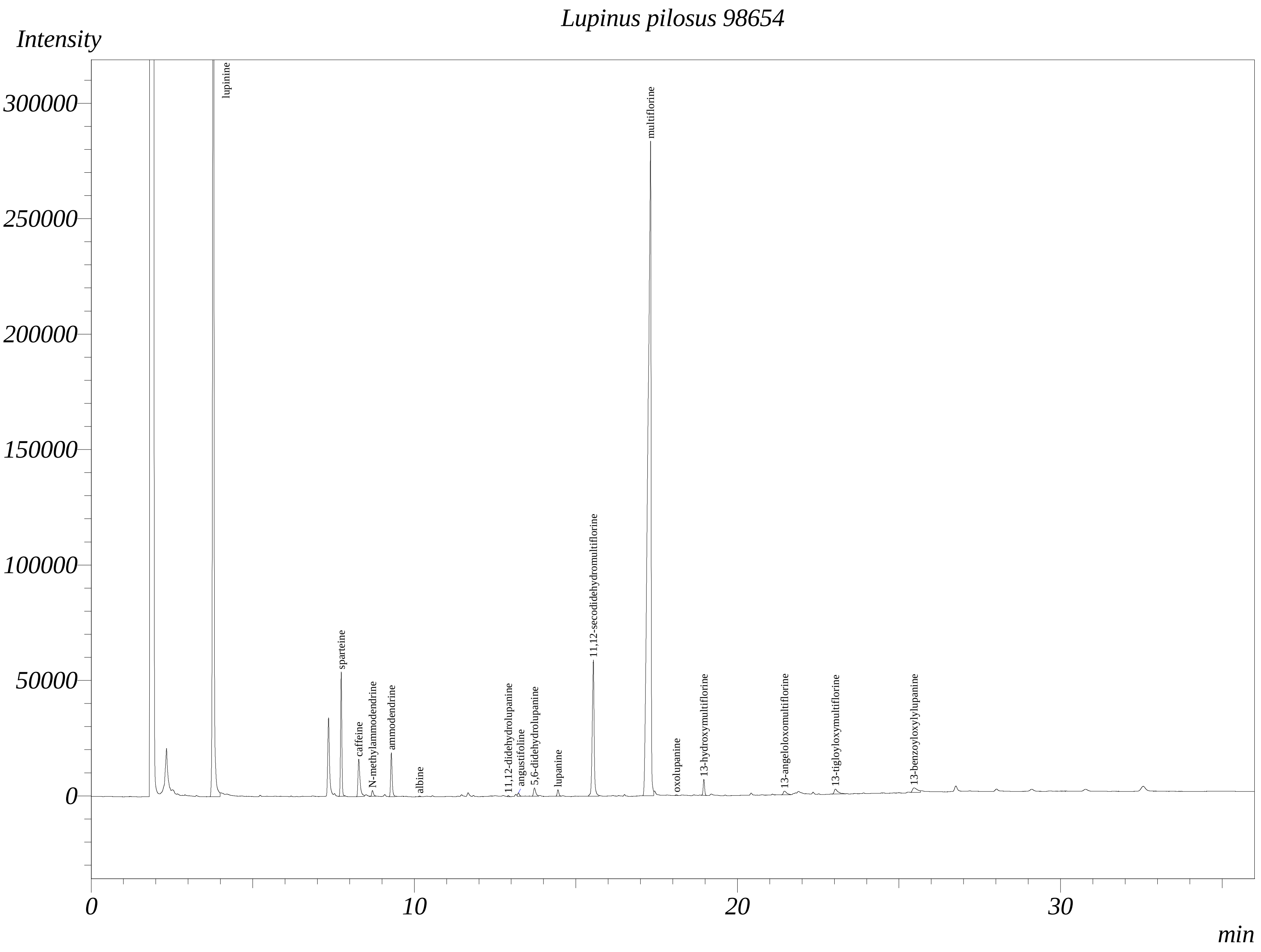

Lupinus pilosus 98654
Intensity
300000
250000
200000
150000
100000
50000
0
lupinine
sparteine
caffeine
N-methylammodendrine
ammodendrine
albine
11,12-didehydrolupanine
angustifoline
5,6-didehydrolupanine
lupanine
11,12-secodidehydromultiflorine
multiflorine
oxolupanine
13-hydroxymultiflorine
13-angeloloxomultiflorine
13-tigloyloxymultiflorine
13-benzoyloxylylupanine
0
10
20
30
min

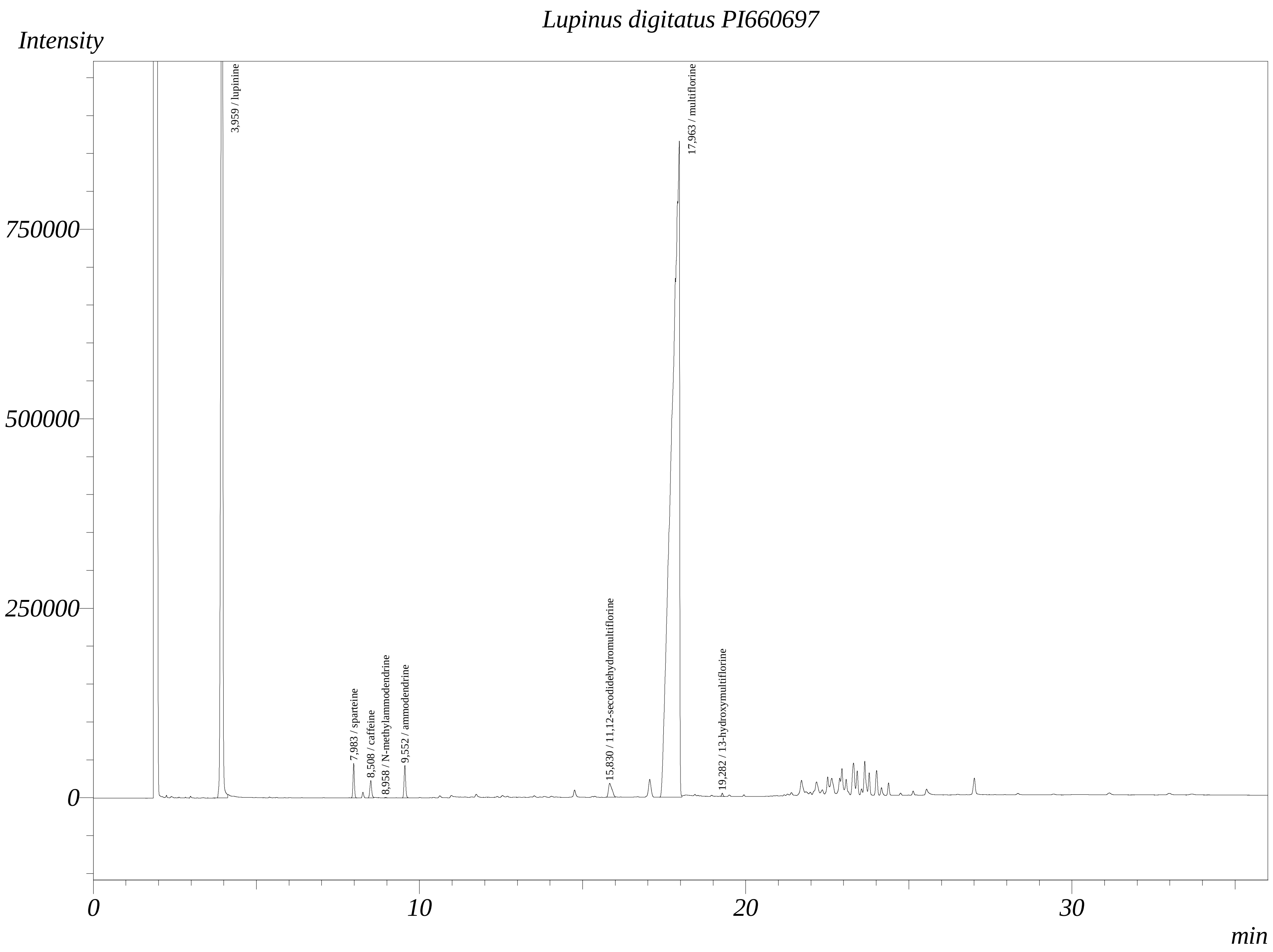
Lupinus digitatus PI660697
Intensity
750000
500000
250000
0
3,959 / lupinine
7,983 / sparteine
8,508 / caffeine
8,958 / N-methylammodendrine
9,552 / ammodendrine
15,830 / 11,12-secodidehydromultiflorine
17,963 / multiflorine
19,282 / 13-hydroxymultiflorine
0
10
20
30
min

Lupinus micranthus 98552

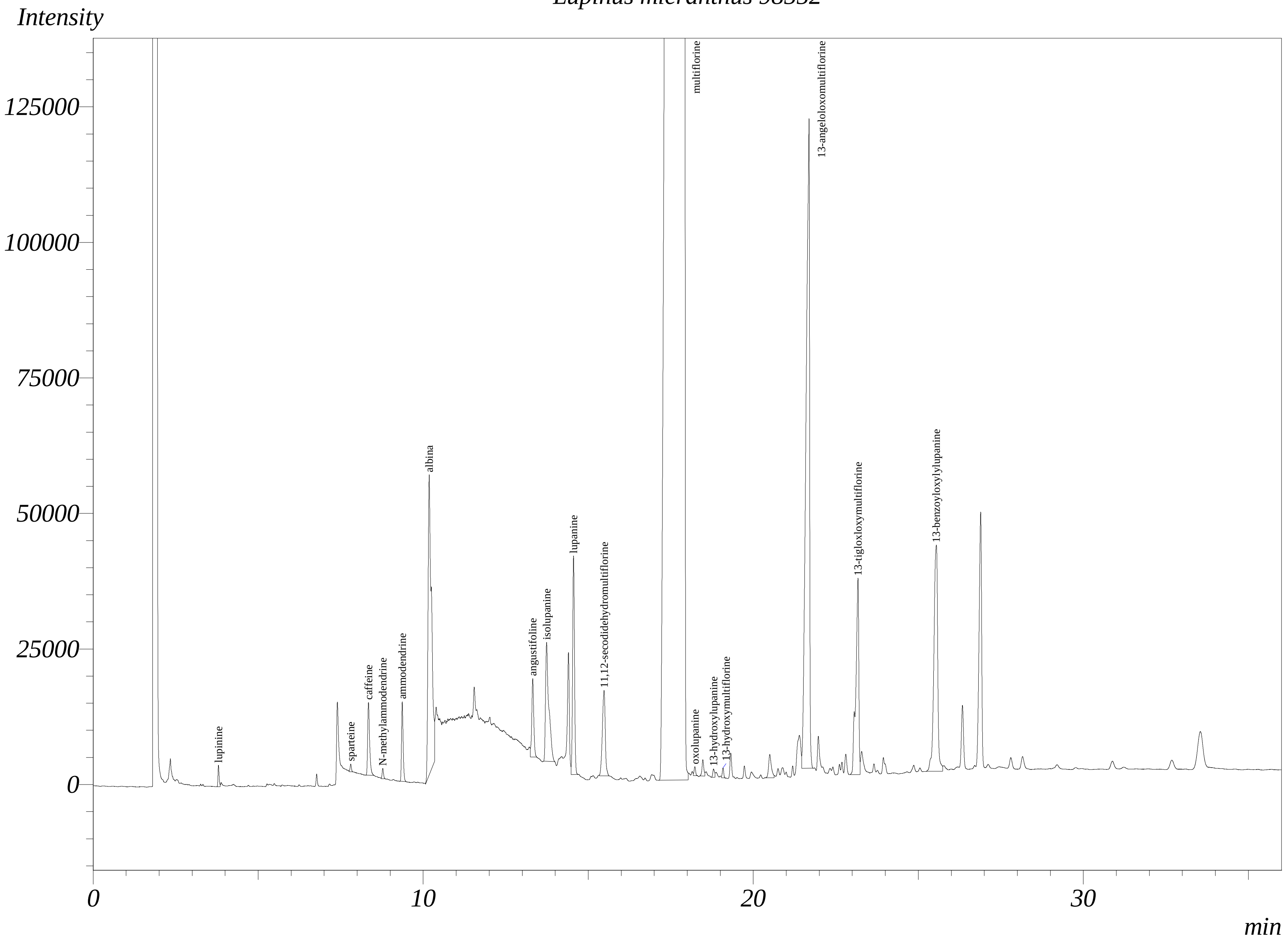

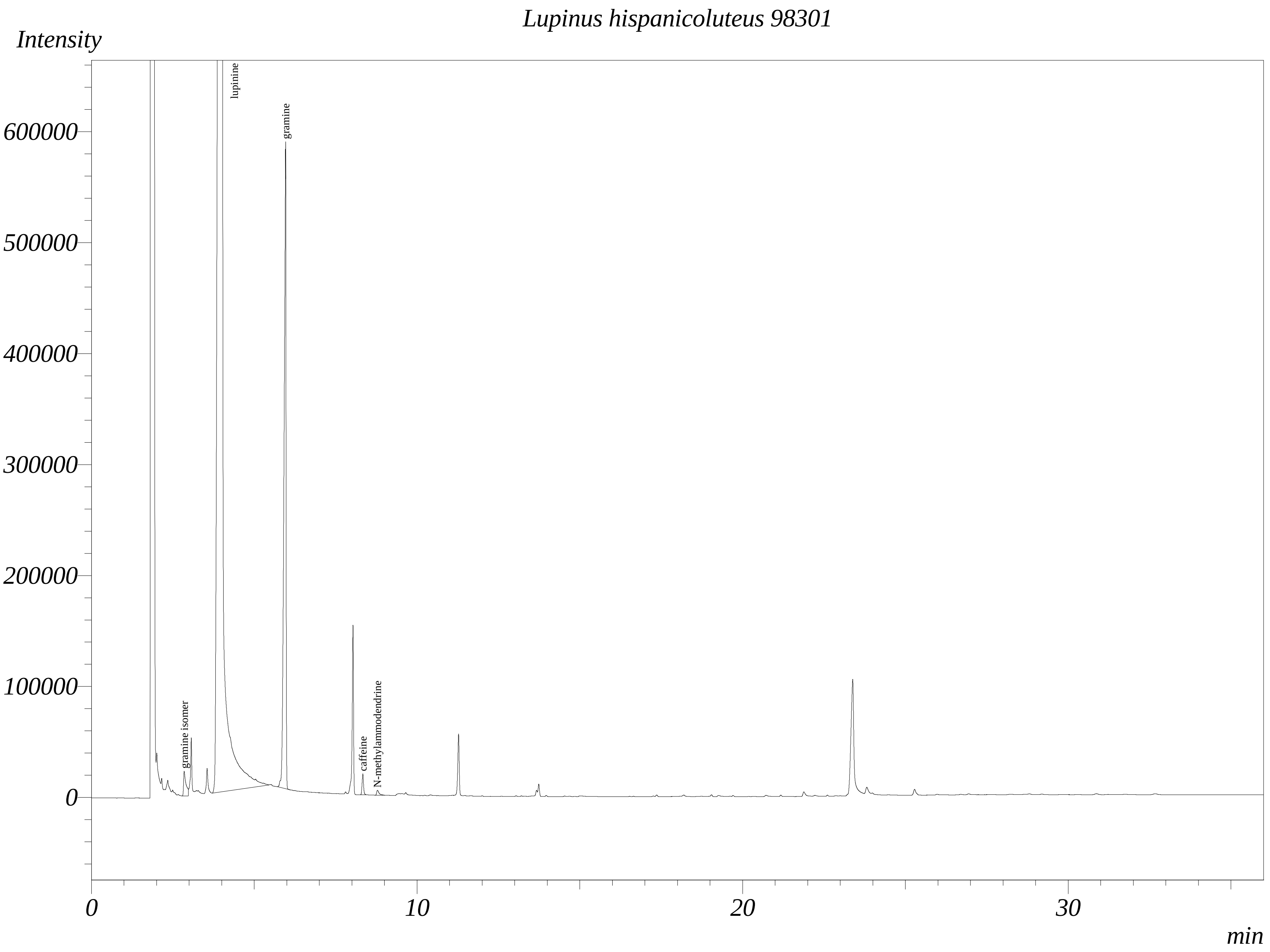

Lupinus hispanicoluteus 98301
Intensity
600000
500000
400000
300000
200000
100000
0
lupinine
gramine
gramine isomer
caffeine
N-methylammodendrine
0
10
20
30
min

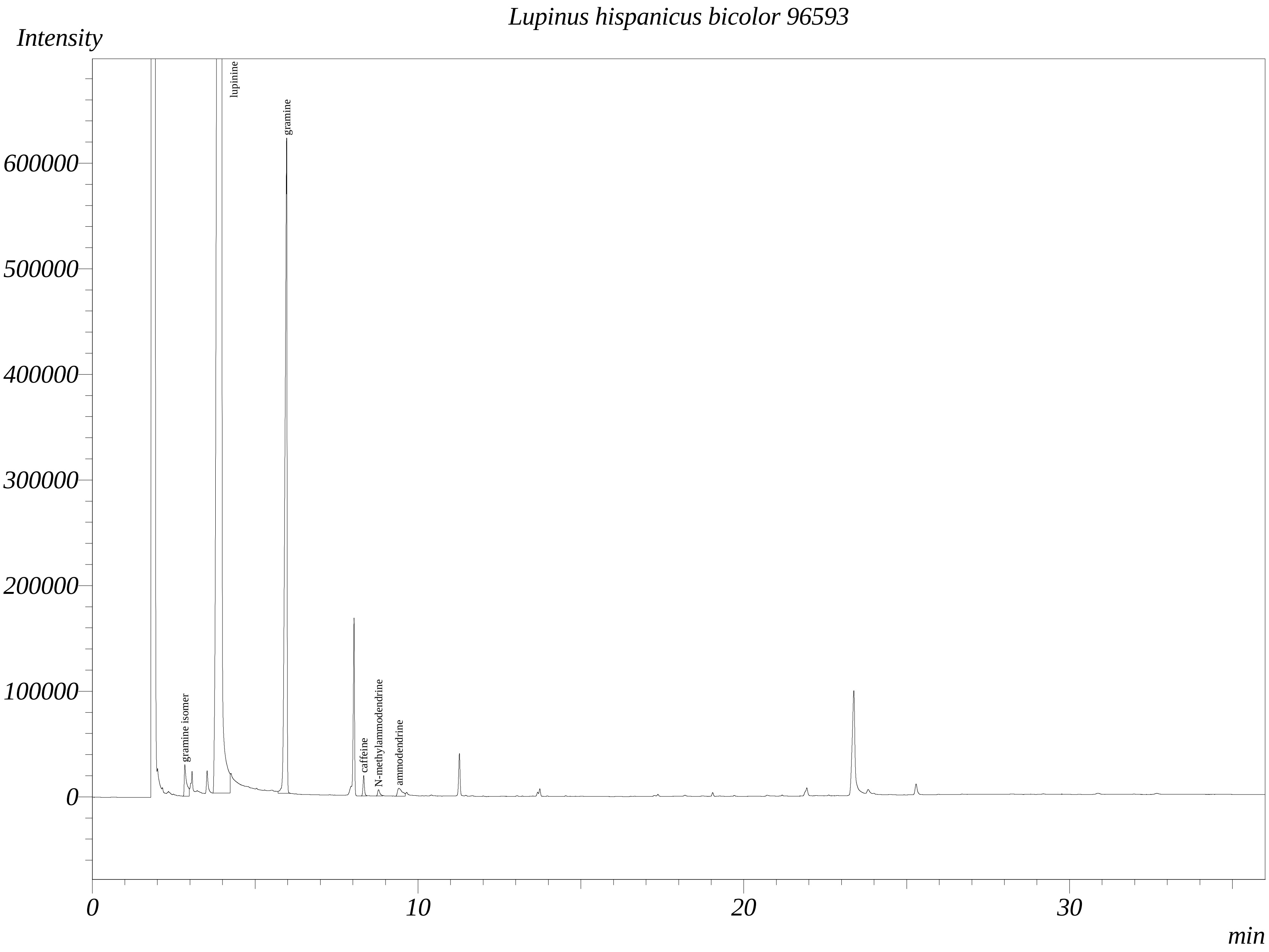
Lupinus hispanicus bicolor 96593
Intensity
600000
500000
400000
300000
200000
100000
0
lupinine
gramine
gramine isomer
caffeine
N-methylammodendrine
ammodendrine
0
10
20
30
min

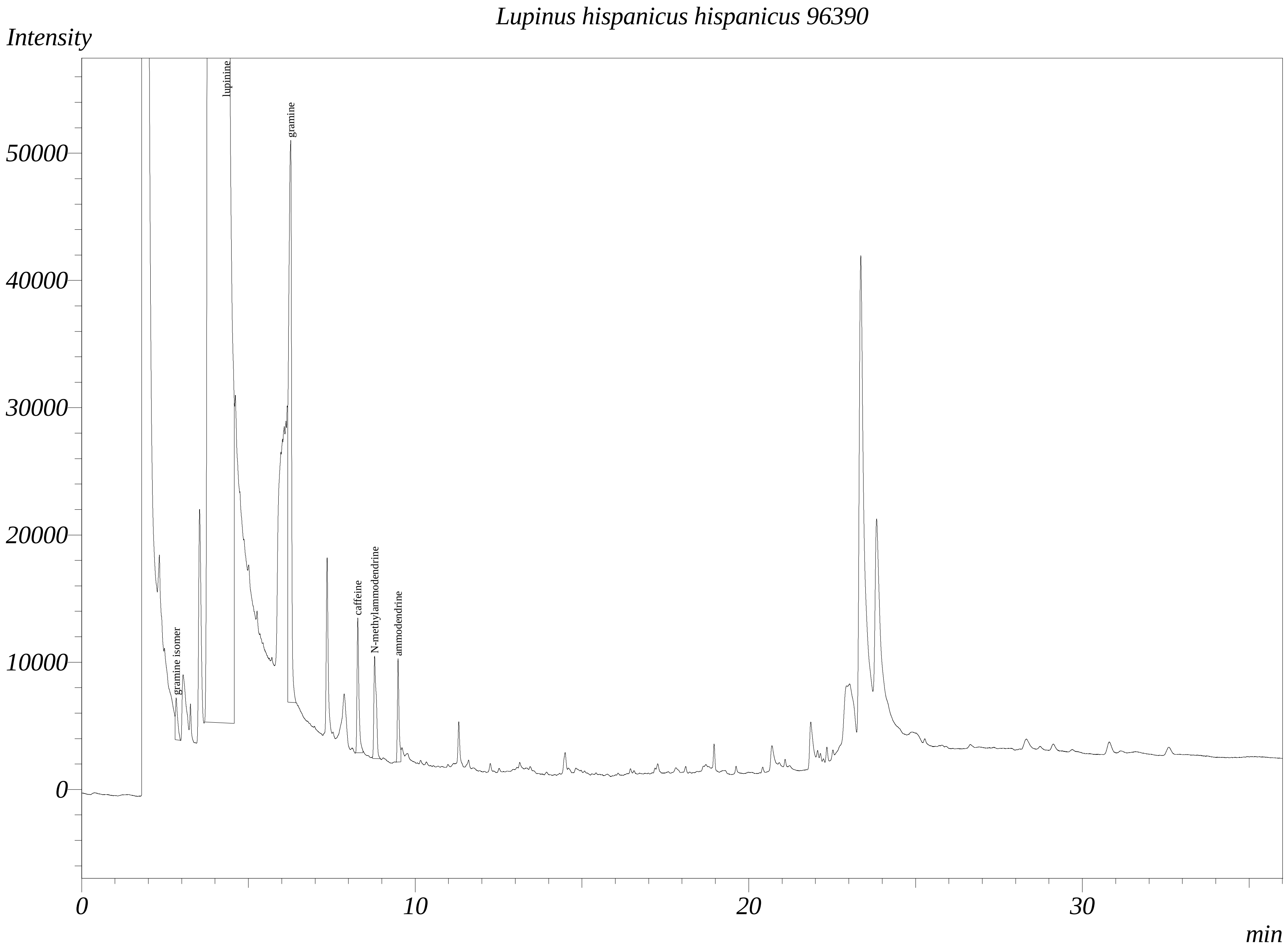
Lupinus hispanicus hispanicus 96390
Intensity
50000
40000
30000
20000
10000
0
lupinine
gramine
gramine isomer
caffeine
N-methylammodendrine
ammodendrine
0
10
20
30
min

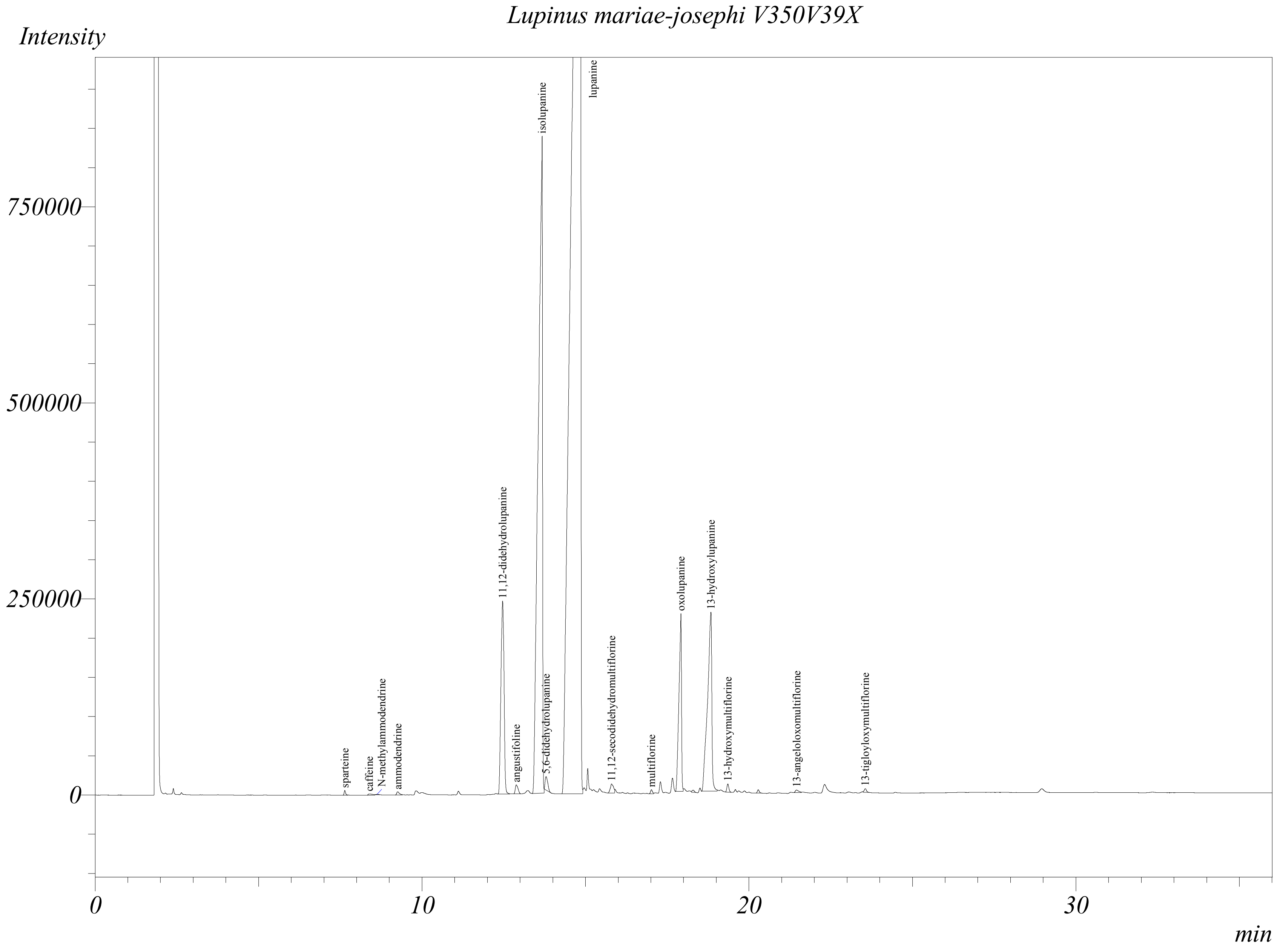

Supplementary Figure S3. Representative chromatograms showing GC separation of the alkaloids from the Old World lupin species. Here the x-axis shows the retention time (min), while y-axis represents signal intensity (pA).
