# Supplementary figures and images for: Chromatographic Fingerprinting of the Old World Lupins Seed Alkaloids: A Supplemental Tool in Species Discrimination
Source: Plants (Basel). 2019 Nov 27;8(12):548. doi: 10.3390/plants8120548 (PMC6963311; doi:10.3390/plants8120548)

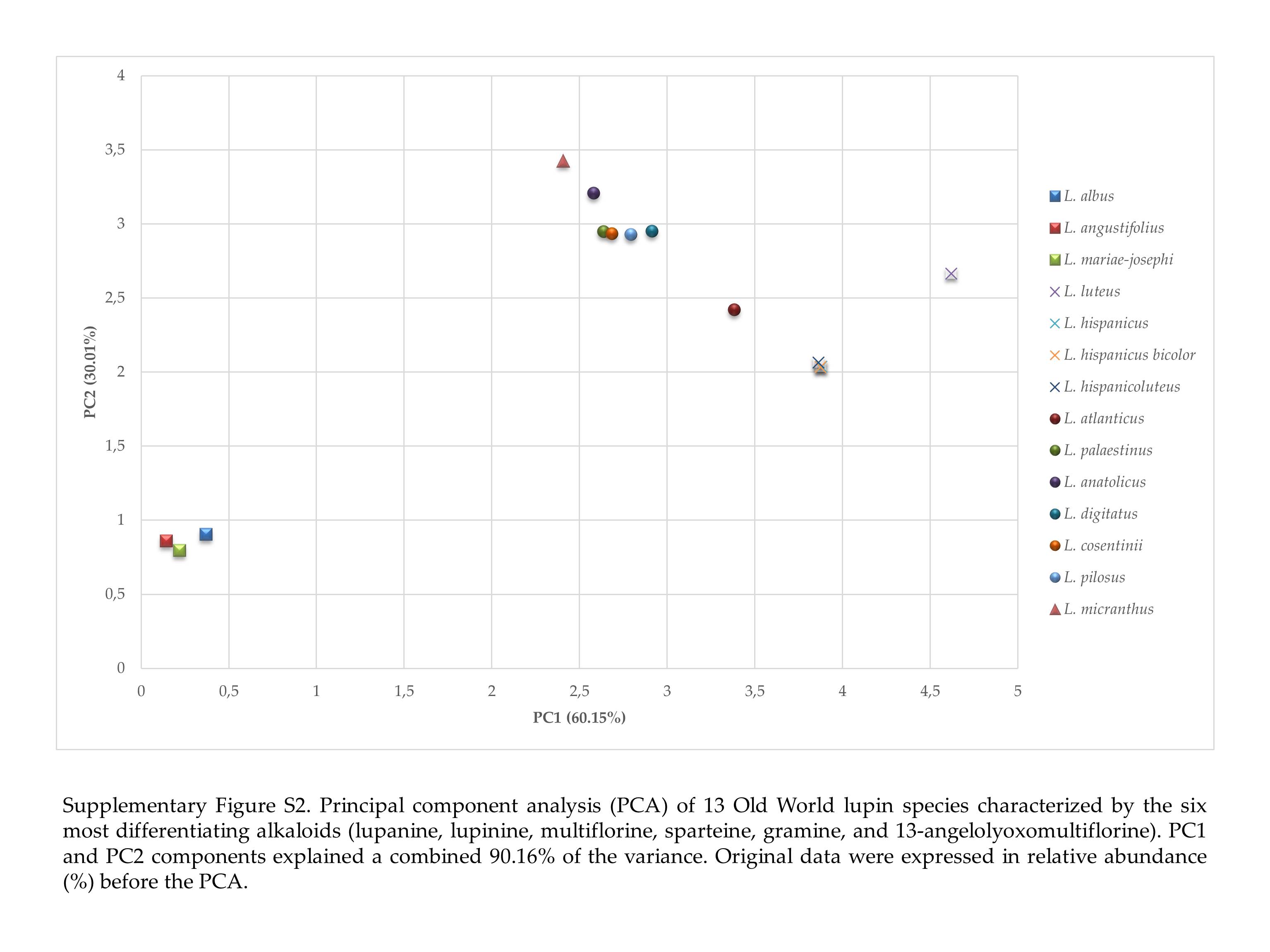

Supplement: Supplementary file 1 [file plants-08-00548-s001.zip › Supplementary Figure S2.jpg]

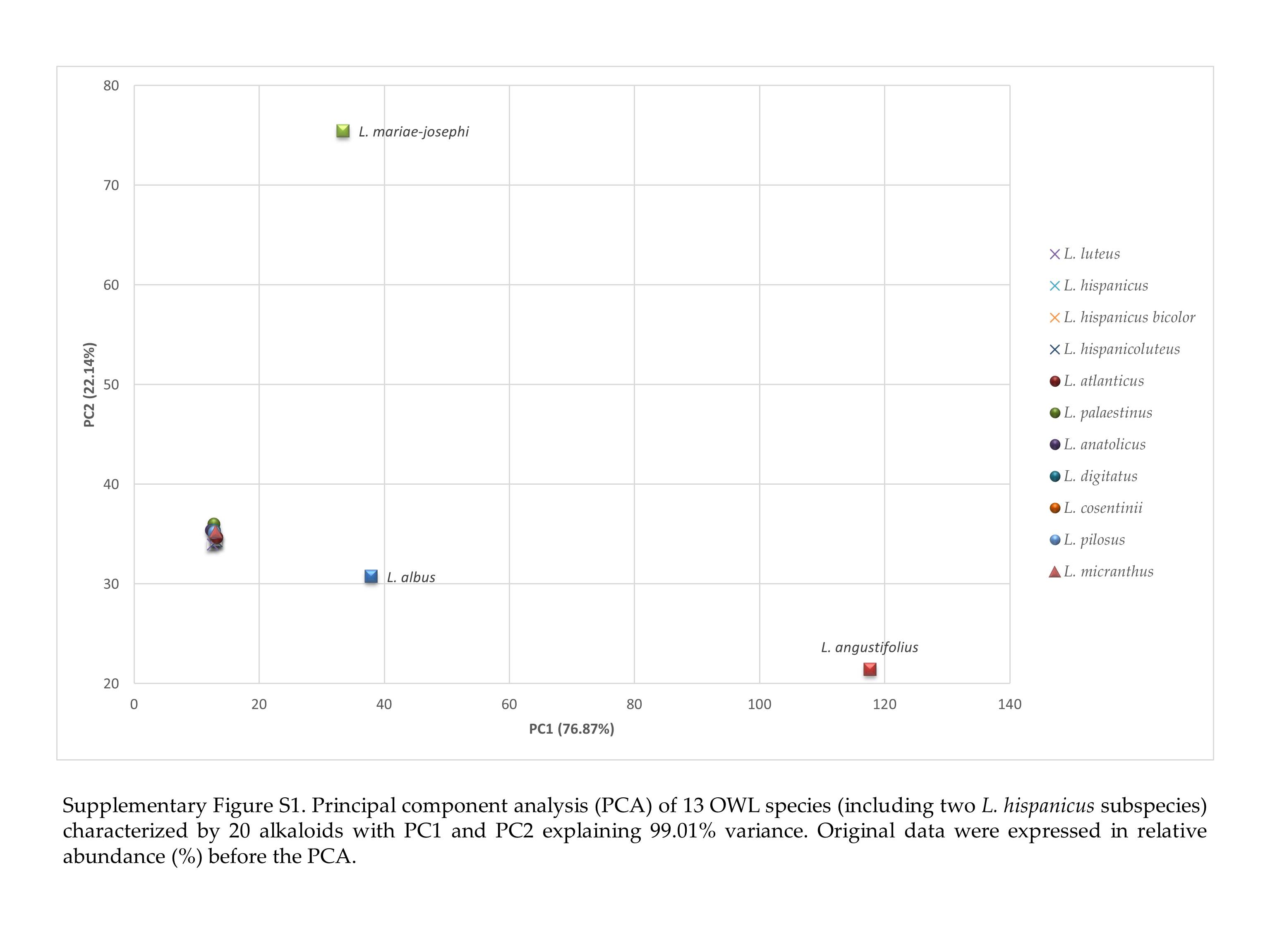

Supplement: Supplementary file 1 [file plants-08-00548-s001.zip › Supplementary Figure S1.jpg]
